# A Copper‐Based Photothermal‐Responsive Nanoplatform Reprograms Tumor Immunogenicity via Self‐Amplified Cuproptosis for Synergistic Cancer Therapy

**DOI:** 10.1002/advs.202500652

**Published:** 2025-03-24

**Authors:** Runzi Cheng, Zhenhao Li, Weican Luo, Hongwu Chen, Tingting Deng, Zhenqi Gong, Qing Zheng, Baizhi Li, Yongming Zeng, Huaiming Wang, Cong Huang

**Affiliations:** ^1^ Department of Gastrointestinal Surgery The First Affiliated Hospital of Shantou University Medical College Shantou 515041 China; ^2^ Shantou University Medical College Shantou 515041 China; ^3^ Southern Medical University Guangzhou 510515 China; ^4^ Department of Neurosurgery The First Affiliated Hospital of Shantou University Medical College Shantou 515041 China; ^5^ Department of Ultrasound The First Affiliated Hospital of Shantou University Medical College Shantou 515041 China

**Keywords:** cuproptosis, elesclomol, immunogenic cell death, photothermally responsive

## Abstract

Studies show that intracellular accumulation of copper ions causes cuproptosis, potentially enhancing anticancer immunity. However, the induction of cuproptosis inevitably faces challenges due to low intracellular copper deliver efficiency and collateral damage to normal tissues. This paper presents a self‐amplified cuproptosis nanoplatform (CEL NP) composed of Cu_2−_ *_X_* S hollow nanospheres (HNSs), elesclomol (ES), and phase‐change material lauric acid (LA). Under NIR‐II laser irradiation, the photothermal energy generated by Cu_2−_ *_X_* S HNSs melts LA, facilitating the precise release of ES and copper ions within the tumor microenvironment. Notably, ES can traverse the cell membrane and form ES‐Cu(II) complexes, thereby enhancing copper delivery within tumor cells. Excess Cu(II) also reacts with endogenous glutathione, reducing its inhibitory effect on cuproptosis. Ultimately, this amplified cuproptosis effect can activate immunogenic cell death, eliciting a robust immune response and promoting tumor suppression. The CEL NP‐mediated release of ES and copper ions offers a novel approach for anticancer therapy through cuproptosis induction.

## Graphical abstract

This study introduces cuproptosis nanoplatform, a novel nanoplatform using Cu_2−_ *_X_* S hollow nanospheres, elesclomol, and lauric acid (LA) for self‐amplified cuproptosis. NIR‐II laser activation melts LA, releasing copper ions and elesclomol to enhance copper delivery within tumor cells, reduce glutathione's inhibitory effect, and trigger immunogenic cell death, thereby improving cuproptosis‐mediated anticancer immunity while minimizing damage to normal tissues.

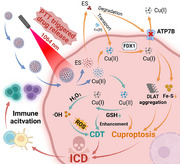

## 1 Introduction

Colorectal cancer (CRC) is a major global health challenge and a leading cause of cancer‐related mortality.^[^ ^1^ ^]^ In CRC treatment, surgery is highly invasive and ineffective against metastasis, while chemotherapy and radiotherapy lack precision and bring significant side effects.^[^ ^2^ ^]^ Immunotherapies targeting programmed cell death protein‐1 (PD‐1) have significantly improved survival rates in patients with CRC, particularly those with microsatellite instability‐high (MSI‐H) tumors.^[^ ^3^ ^]^ A key resistance mechanism in CRC immunotherapy is insufficient presence of tumor‐infiltrating lymphocytes (TILs) in the tumor microenvironment (TME).^[^ ^4^ ^]^ Emerging research suggests that cuproptosis is a novel therapeutic approach for cancer.^[^ ^5^ ^]^ This process involves the excessive uptake of Cu(II), which is subsequently reduced to Cu(I) by ferredoxin 1 (FDX1) within mitochondria. Cu(I) then binds to lipoylated dihydrolipoamide S‐acetyltransferase (DLAT), leading to its abnormal aggregation. In addition, an overabundance of Cu(I) downregulates the expression of iron‐sulfur (Fe‐S) cluster proteins, leading to proteotoxic stress and cell death.^[^ ^6^ ^]^ Notably, numerous studies have confirmed that cuproptosis can induce immunogenic cell death (ICD), enhancing the infiltration of immune‐related cells, such as dendritic cells (DCs), CD8^+^, and CD4^+^ T cells.^[^ ^7^ ^]^ This immune activation is facilitated by the release of damage‐associated molecular patterns (DAMPs) from dying tumor cells, including calreticulin (CRT), high mobility group box 1 (HMGB1), and adenosine triphosphate (ATP).^[^ ^8^ ^]^ Ultimately, the cuproptosis‐inducing ICD effect may sensitize CRC cells to immunotherapy.^[^ ^9^ ^]^

Despite the potential of copper overload to induce cuproptosis, a series of regulatory mechanisms maintain copper ion homeostasis within cells, ensuring that intracellular copper concentrations remain relatively stable.^[^ ^10^ ^]^ Researchers have found that when cells are exposed to high levels of copper ions, Cu‐ATPases (ATP7A/B) facilitate the efflux of copper ions across cell membranes to maintain homeostasis.^[^ ^11^ ^]^ Therefore, merely increasing the external supply of copper ions is scarcely adequate to trigger cuproptosis effectively. Encouragingly, recent studies have revealed that elesclomol (ES), traditionally used to induce oxidative stress, also functions as a copper ionophore. ES can form a stable 1:1 complex with Cu(II) outside the cell, shuttle Cu(II) across cellular membranes, and deliver it to the mitochondria.^[^ ^12^ ^]^ Moreover, ES can degrade Cu‐ATPases in CRC cells, further disrupting copper homeostasis within tumor cells.^[^ ^13^ ^]^ Thus, combining ES with an external supply of copper ions is expected to effectively induce cuproptosis. However, the lack of targeting specificity of ES, along with its rapid clearance and metabolism in the bloodstream limit its therapeutic effects and increase the risk of damage to normal tissues, restricting its clinical use.^[^ ^14^ ^]^ Therefore, achieving precise, simultaneous and responsive targeted release of ES and copper ions within tumor cells is crucial for enhancing copper ion delivery and amplifying the cuproptosis effect for cancer therapy.^[^ ^15^ ^]^

Another barrier to cuproptosis induction is the overexpression of glutathione (GSH) in tumor cells.^[^ ^16^ ^]^ GSH can bind to excess Cu(I), forming GSH‐Cu complexes that prevent Cu(I) from triggering cuproptosis in mitochondria.^[^ ^17^ ^]^ Interestingly, Cu(II) can deplete GSH through reduction reactions, generating Cu(I), which not only binds to ferredoxin 1 (FDX1) to promote cuproptosis but also catalyzes Fenton‐like reactions in the slightly acidic TME.^[^ ^18^ ^]^ Recently, Cu(II)‐containing nanoparticles (NPs) have emerged as promising reservoirs for copper ions in inducing cuproptosis. Among these, Cu_2−_ *_X_* S hollow nanospheres (HNSs) have demonstrated the ability to release copper ions in response to the weakly acidic pH of TME, with their high specific surface area further enhancing this release.^[^ ^19^ ^]^ Moreover, due to their superior photothermal conversion efficiency in the second near‐infrared (NIR‐II) window, Cu_2−_ *_X_* S HNSs are ideal for photothermal‐responsive drug delivery systems.^[^ ^20^ ^]^

**Scheme 1:**
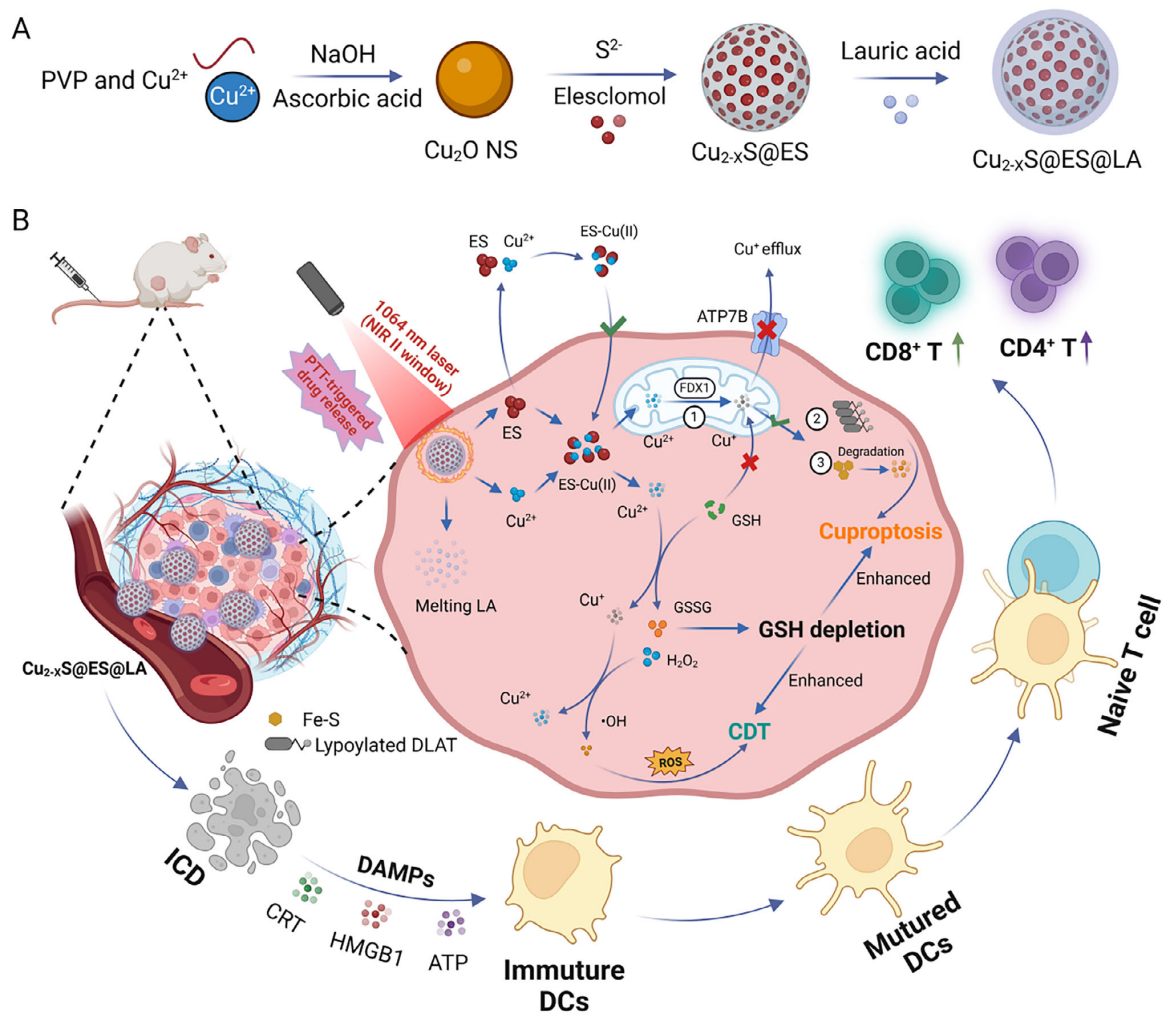
Schematic illustration of the NIR‐II photothermal‐triggered cuproptosis effect of CEL NP. A) Synthesis process of CEL NP. B) Mechanism of the therapeutic effect of amplified cuproptosis combined PTT/CDT and the activation of the immune response (Schematics were created with BioRender.com).

In this study, we developed an innovative nanoplatform for enhanced cuproptosis induction, termed Cu_2−_ *_X_* S@Elesclomol@LA (CEL NP) (**Scheme** **1**). This platform was synthesized by encapsulating ES within Cu_2−_ *_X_* S HNSs, followed by coating with phase‐change lauric acid (LA, melting point:44–46 °C) to reduce premature ES release and minimize the side effects on normal tissues during drug transit. Upon NIR‐II irradiation, the heat generated by Cu_2−_ *_X_* S HNSs not only induces tumor cell damages via photothermal therapy (PTT), but also melts LA, causing an explosive release of ES and copper ions within the TME. The released ES forms ES‐Cu(II) complexes, increasing the mitochondrial Cu(II) concentration in cancer cells. Meanwhile, the dynamic interaction between Cu(II)/Cu(I) from CEL NP and GSH depletion enhances cuproptosis and promotes the production of cytotoxic reactive oxygen species (ROS). ^[^ ^21^ ^]^ Ultimately, the amplified cuproptosis effect, combined with PTT and chemodynamic therapy (CDT), could trigger ICD and reshape the immunosuppressive TME, offering a novel approach for CRC treatment and potentially improving cancer immunotherapy outcomes.

## 2 Results and Discussion

### 2.1 Synthesis and Characterization of CEL NP

**Figure 1:**
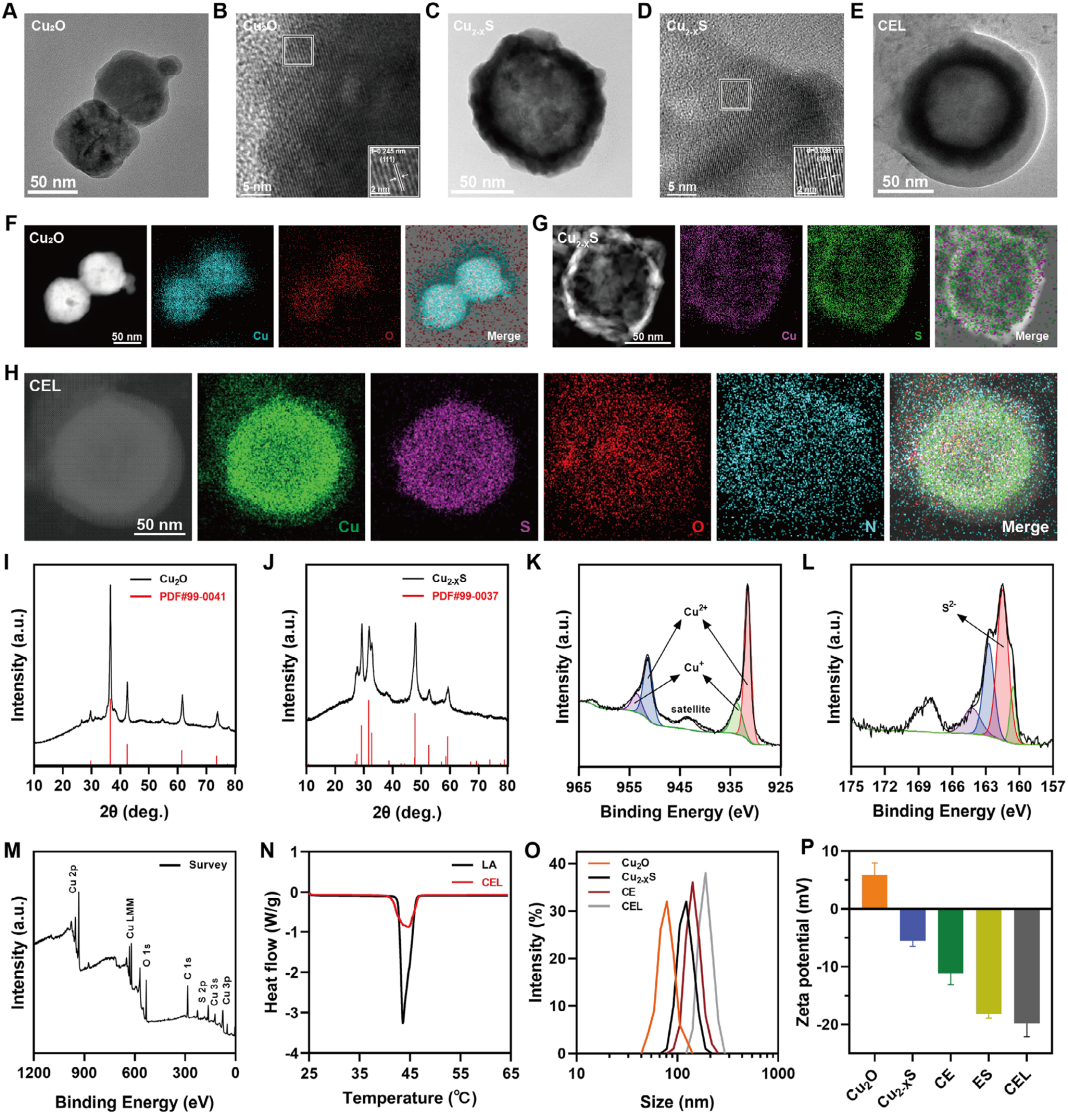
Physiochemical characterizations of CEL NP. TEM and HRTEM images of A,B) Cu_2_O NSs and C,D) Cu_2−_ *_X_* S HNSs; E) TEM image of CEL NP; EDS mapping images of F) Cu_2_O NSs, G) Cu_2−_ *_X_* S HNSs, and H) CEL; XRD patterns of I) Cu_2_O NSs and J) Cu_2−_ *_X_* S HNSs; K) Cu 2p and L) S 2p XPS spectra of Cu_2−_ *_X_* S HNSs; M) XPS survey spectrum of Cu_2−_ *_X_* S HNSs; N) DSC curves of LA and CEL NP; O) average diameters and P) zeta potentials of different nanoparticles (*n* = 3). Data are presented as mean ± SD.

In the first step, Cu_2_O nanospheres (NSs) were synthesized by introducing ascorbic acid (AA) into a suspension containing copper ions, NaOH, and the capping agent PVP. Transmission electron microscopy (TEM) and scanning electron microscopy (SEM) analyses showed that the Cu_2_O NSs were uniformly spherical with an average diameter of ≈70 nm (**Figure** **1**A; Figure S1A, Supporting Information). High‐resolution TEM (HRTEM) images revealed a lattice spacing of ≈0.245 nm, corresponding to the (222) interplanar spacing of Cu_2_O nanoparticles (Figure 1B). X‐ray diffractometry (XRD) analysis confirmed the phase of the Cu_2_O NSs, with diffraction peaks matching those of standard spherical Cu_2_O (JCPDS No. 99‐0041) (Figure 1I). In addition, the energy‐dispersive spectroscopy (EDS) and EDS mapping demonstrated a homogeneous distribution of Cu and O elements, confirming the purity of the synthesized Cu_2_O NSs without detectable impurities (Figure 1F; Figure S2A, Supporting Information).

Cu_2−_ *_X_* S HNSs were synthesized using the sacrificial template method.^[^ ^22^ ^]^ TEM and SEM analyses revealed an increase in size to ≈110 nm for Cu_2−_ *_X_* S HNSs, potentially due to the “Kirkendall effect,” in which the different diffusion rates of S and O create voids during the formation of Cu_2−_ *_X_* S on the surface (Figure 1C; Figure S1B, Supporting Information).^[^ ^23^ ^]^ HRTEM analysis confirmed a lattice spacing of ≈0.329 nm, corresponding to the (111) interplanar spacing of spherical Cu_2−_ *_X_* S (Figure 1D). XRD analysis verified that Cu_2−_ *_X_* S HNSs were consistent with standard spherical Cu_2−_ *_X_* S (JCPDS No. 99‐0037) (Figure 1J). EDS and EDS mapping showed that the Cu_2−_ *_X_* S HNSs exhibited a high level of purity, with a hollow core and a surrounding shell layer (Figure 1G; Figure S2B, Supporting Information). X‐ray photoelectron spectroscopy (XPS) analysis of the Cu_2−_ *_X_* S HNSs identified the presence of C 1s, O 1s, S 2p, and Cu 2p in the survey spectra (Figure 1M).^[^ ^24^ ^]^ A core‐level peak at 951.53 eV and a satellite peak at 933.73 eV in the Cu 2p3/2 region indicated that the valence states of Cu in the Cu_2−_ *_X_* S HNSs included both Cu(I) and Cu(II) (Figure 1K).^[^ ^24^ ^]^ The Cu LMM core‐level spectra further confirmed the presence of Cu(I) in Cu_2−_ *_X_* S HNSs (Figure S3, Supporting Information).^[^ ^24^ ^]^ In addition, the S 2p peak at 161.48 eV confirmed the presence of S(II) in the Cu_2−_ *_X_* S HNSs (Figure 1L).^[^ ^24^ ^]^ Overall, these material characterizations confirmed the successful synthesis of Cu_2−_ *_X_* S HNSs.

Elesclomol (ES) functions as a micromolecular copper ionophore, capable of forming stable ES‐Cu complexes.^[^ ^25^ ^]^ To assess the drug‐loading capacity of Cu_2−_ *_X_* S HNSs, Cu_2−_ *_X_* S@Elesclomol nanoparticles were prepared by dispersing Cu_2−_ *_X_* S HNSs in an ES DMSO solution overnight. The UV–vis–NIR spectra of ES exhibited a broad characteristic peak at 380 nm (Figure S4A, Supporting Information), with a linear correlation between ES concentration and absorbance (Figure S4B, Supporting Information). Compared with ES DMSO solution, a decrease in absorbance at 380 nm in the supernatant of Cu_2−_ *_X_* S@elesclomol nanoparticles confirmed successful ES loading, with a capacity of 6.40% (Figure S4C, Supporting Information). To confer thermal responsiveness to the nanoplatform, LA, known for its biocompatibility and biodegradability, was used to modify Cu_2−_ *_X_* S@Elesclomol, forming Cu_2−_ *_X_* S@Elesclomol@LA (CEL NP).^[^ ^26^ ^]^ TEM images confirmed the spherical structure of the CEL NP with an increased size (Figure 1E). EDS mapping of the CEL NP revealed a similar hollow distribution of Cu and S elements, while O and N elements showed a uniform distribution, confirming the successful assembly of ES on the Cu_2−_ *_X_* S HNSs (Figure 1H). Differential scanning calorimetry (DSC) analysis indicated similar melting points for CEL NP and LA, verifying successful surface LA surface modification of Cu_2−_ *_X_* S@Elesclomol (Figure 1N).^[^ ^27^ ^]^ In addition, dynamic light scattering (DLS) analysis showed a progressive increase in the hydrodynamic sizes of Cu_2_O (78.82 nm), Cu_2−_ *_X_* S (122.41 nm), Cu_2−_ *_X_* S@Elesclomol (141.77 nm), and CEL NP (190.14 nm) (Figure 1O). The negative zeta potential of the CEL NP after LA coating enhanced its circulatory stability (Figure 1P). The appropriate size (100–200 nm) and negative surface charge of CEL NP could support their enhanced accumulation at tumor sites through the enhanced permeability and retention (EPR) effect.^[^ ^28^ ^]^

### 2.2 Evaluation of the NIR‐II Photothermal Effect and Photothermal‐Enhanced ROS Production by CEL NP

**Figure 2:**
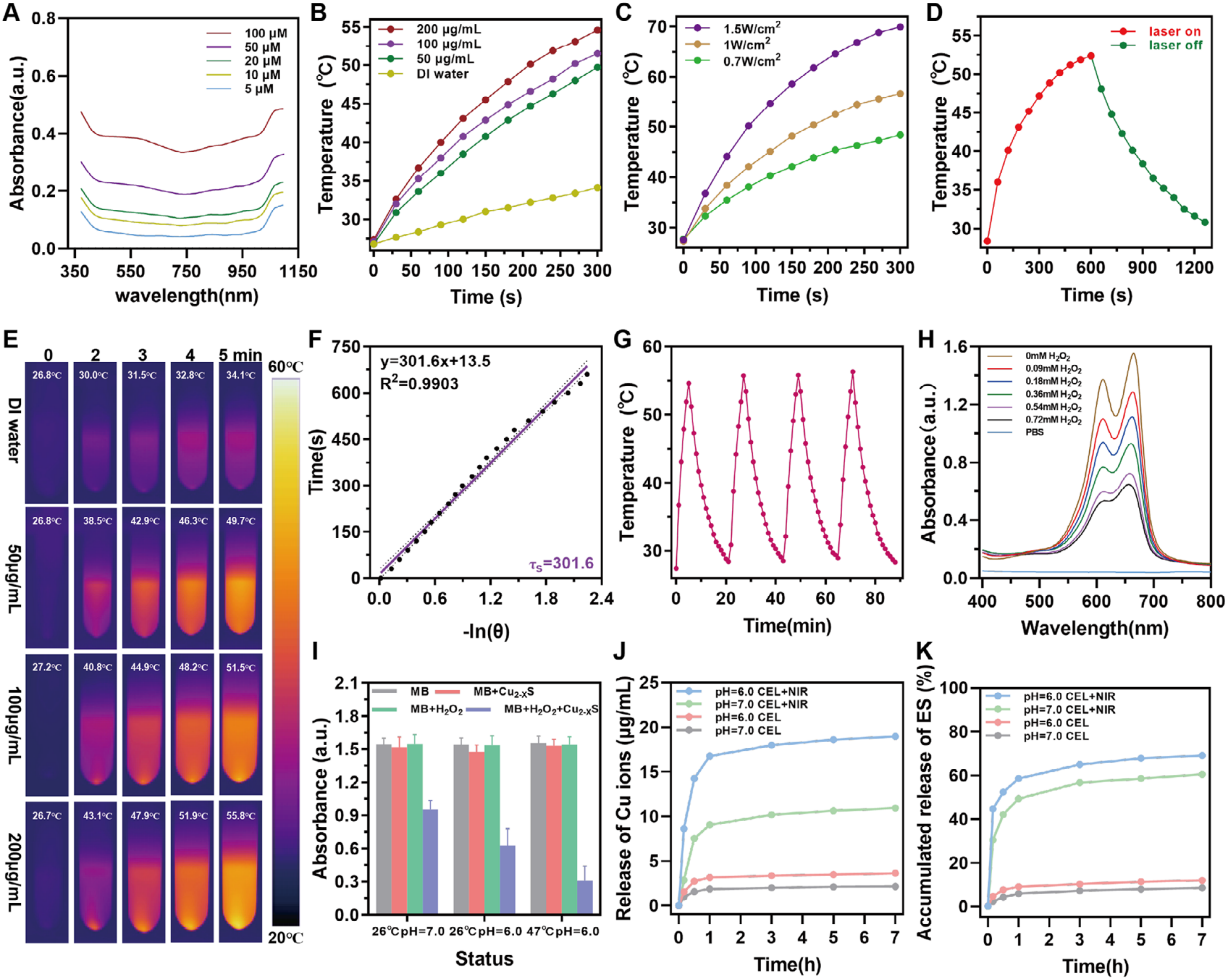
Photothermal performance and Fenton‐like properties of CEL NP. A) UV–vis–NIR spectra of CEL NP at different concentrations. Photothermal heating curves of CEL NP at B) different concentrations and at C) different laser powers. D) Photothermal irradiation and natural cooling curve of the CEL solution. E) Photothermal images of CEL NP at different concentrations under 1 W cm^−2^. F) Linear relationship between time and ln(*θ*) during the cooling period. G) Temperature variations during four on/off cycles of laser irradiation (1064 nm, 1 W cm^−2^, for 5 min). H) Fenton‐like properties of Cu_2‐X_S HNSs via MB probe in the presence of H_2_O_2_ (0–0.72 × 10^−3^ m). I) Statistical analysis of MB degradation under different experimental conditions (*n* = 3). Release of J) copper ions and K) ES from CEL NP with and without laser irradiation (1064 nm, 1 W cm^−2^, for 5 min). Data are presented as mean ± SD.

The photothermal properties of nanoparticles rely on their strong absorption in the NIR window.^[^ ^29^ ^]^ As shown in the UV–vis–NIR spectra, CEL NP exhibited increasing absorption in the NIR‐II (≥1000 nm) region (**Figure** **2**A), enabling the generation of thermal energy for cancer treatment and facilitating the melting of LA under NIR‐II laser irradiation. Compared with the NIR‐I (700–900 nm) window, the NIR‐II (1000–1700 nm) window offers greater tissue penetration depth and a higher signal‐to‐noise ratio (SBR), making it more suitable for deep tissue therapy.^[^ ^30^ ^]^ The photothermal performance of CEL NP was evaluated using a thermometer and an infrared thermal camera. As depicted in Figure 2E, thermal images showed that the temperature of CEL NP (200 µg mL^−1^) increased from 26.7 to 55.8 °C upon exposure to a 1064 nm laser (1 W cm^−2^, for 5 min), which were significantly higher than the increase observed in PBS under the same conditions (Δ*T* = +7 °C). Similar to other photothermal agents, the thermal energy produced by CEL NP was dependent on its concentration, the laser power density and the irradiation duration (Figure 2B,C). The photothermal conversion efficiency (*η*) of CEL NP was calculated based on the published literature.^[^ ^31^ ^]^ The solution temperature was recorded during both heating and natural cooling phases (Figure 2D). The time constant *τ*_s_ was calculated from the slope of the linear fit to the cooling data (*t* vs ln*θ*) (Figure 2F). Using the relevant formulas and data, the photothermal conversion efficiency was determined to be 37.2%, comparable to other cupro‐sulfur‐based nanoparticles, such as H‐Cu_9_S_8_/PEG NPs (40.9%),^[^ ^32^ ^]^ PS@Cu_9_S_8_ nanocatalysts (42.34%),^[^ ^33^ ^]^ and CuS hollow nanoflowers (30%).^[^ ^34^ ^]^ Importantly, no significant decrease in the peak temperature of the Cu_2−_ *_X_* S solution was observed during four NIR‐II laser on/off irradiation cycles, confirming the excellent photothermal stability of CEL NP (Figure 2G).

Moreover, the heat generated by PTT can promote the Fenton‐like reaction involving Cu(I), thereby enhancing Cu(I)‐mediated CDT.^[^ ^35^ ^]^ To evaluate the Fenton‐like properties of the Cu_2‐X_S HNSs, ROS (·OH) was assessed through methylene blue (MB) degradation.^[^ ^36^ ^]^ As the concentration of H_2_O_2_ increased, the characteristic MB absorption peak at 664 nm progressively decreased, indicating that Cu_2−_ *_X_* S HNSs could potentially generate ROS in the presence of intracellular H_2_O_2_ (Figure 2H; Figure S5A, Supporting Information). Furthermore, elevating the reaction temperature to 47 °C to simulate the temperature increase during PTT further enhanced the efficiency of ·OH production (Figure 2I; Figure S5B, Supporting Information). Thus, the elevation in temperature induced by photothermal energy, combined with a mildly acidic environment, can significantly boost the generation of ROS through the CDT facilitated by Cu_2−_ *_X_* S HNSs.^[^ ^18b^ ^]^

The drug release capacity of CEL NP in response to photothermal stimulation was examined. LA, used as a surface modifier, belongs to the family of phase‐change materials (PCMs) and serves as an intelligent matrix for temperature‐triggered release.^[^ ^37^ ^]^ With a melting point of 44–46 °C, LA remains solid at physiological temperature (37 °C), effectively preventing the premature release of ES. However, when the local photothermal energy exceeds the melting point of LA (>46 °C), it rapidly transitions into a transparent liquid phase, enabling ES to facilitate the transfer of Cu(II) into tumor cells. This enhances the targeted delivery of cytotoxic drugs to tumor sites.^[^ ^38^ ^]^ The release of copper ions was measured using inductively coupled plasma‐mass spectrometry (ICP‐MS). Initial observations showed that copper ion release from the Cu_2−_ *_X_* S solution increased under acidic and photothermal condition (1064 nm, 1 W cm^−2^, for 5 min) (Figure S6, Supporting Information). Further evaluation of CEL NP revealed that at 37 °C and pH 7.0, only 2.12 µg mL^−1^ of copper ions were released over 7 h, indicating that the LA coating effectively prevented Cu_2−_ *_X_* S from interacting with the weakly acidic environment (Figure 2J). However, when the temperature was increased above 46 °C under laser irradiation (1064 nm, 1 W cm^−2^, for 5 min), copper ion release was significantly enhanced due to the melting of LA and the increased solubility product constant (*K*_sp_) of the Cu_2−_ *_X_* S solution.^[^ ^39^ ^]^ Under a weakly acidic pH of 6.0, the released copper ion concentration peaked at 18.60 µg mL^−1^ within 7 h. Likewise, ES release was quantified by measuring the characteristic UV–vis–NIR absorption peak at 380 nm. In the absence of irradiation, ES release was limited to 11%; however, under irradiation at pH 6.0, it surged dramatically, achieving a release efficiency of 69% after 7 h (Figure 2K). In conclusion, the combination of laser irradiation and acidic stimulation effectively triggers rapid release of both copper ions and ES from the CEL NP, enhancing their therapeutic potential.

#### 2.2.1 In Vitro Evaluation of Synergistic Amplified‐Cuproptosis Combined with the PTT/CDT Antitumor Effect

**Figure 3:**
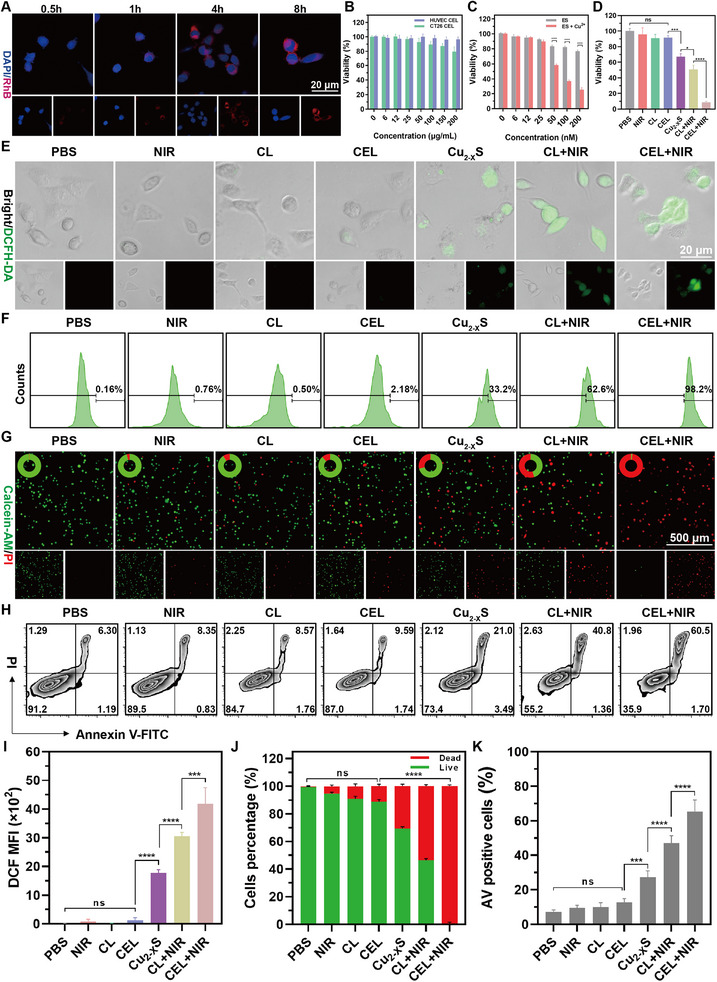
In vitro evaluation of the synergistic antitumor effects of CEL NP. A) Fluorescence images showing the intracellular uptake of CEL‐RhB by CT26 cells. B) Cell viability of HUVEC and CT26 cells treated with different concentrations of CEL NP using the CCK‐8 assay (*n* = 5). C) Cell viability of CT26 cells incubated with ES or ES+Cu^2+^ treatments (*n* = 5). D) CT26 Cell viability following various treatment conditions: PBS, NIR, CL, CEL, Cu_2−_ *_X_* S, CL+NIR, and CEL+NIR ([Cu]: 25 µg mL^−1^, NIR: 1064 nm, 1 W cm^−2^, for 8 min) (*n* = 5). E) CLSM images illustrating ROS production in CT26 cells treated with different therapies. F) FCM analysis and I) its quantification of ROS production from (E) (*n* = 3). G) Fluorescence images and J) quantitative analysis of live/dead cells percentages of Calcein‐AM/PI co‐dyed CT26 cells treated with different groups. H) FCM data and K) statistical analysis of the AV positive rate for CT26 cells treated with different groups (*n* = 3). Data are presented as mean ± SD. *p*‐values were calculated by one‐way ANOVA test. *p < 0.05, **p < 0.01, ***p < 0.001, ****p < 0.0001, ns, not significant.

To evaluate the intracellular uptake of CEL NP, we tagged them with rhodamine B (RhB). CLSM images showed a time‐dependent increase in intracellular uptake efficiency, with a prominent fluorescent signal detected after 4 h of incubation (**Figure** **3**A). This trend was further confirmed by flow cytometry (FCM), revealing that the internalization rate of CEL NP by CT26 cells rose progressively with extended incubation time (Figure S7, Supporting Information). To evaluate the antitumor effects of CEL NP, we assessed the biocompatibility of CEL NP, Cu_2−_ *_X_* S HNSs, and ES in both HUVEC and CT26 cells using the CCK‐8 assay. The results showed that both ES and CEL NP displayed excellent biocompatibility (Figure 3B; Figure S8A, Supporting Information). In contrast, bare Cu_2−_ *_X_* S HNSs (25–200 µg mL^−1^) reduced cell viability in both cell lines, likely due to the absence of LA modification (Figure S8B, Supporting Information). The synergistic antitumor effects of CEL NP under irradiation were then assessed. As expected, the cell‐killing efficacy of the CL+NIR treatment was dependent on both drug dosage and laser exposure time (Figure S8C, Supporting Information). While ES alone, functioning as a copper ionophore, showed no significant cytotoxicity when incubated with CT26 cells, it exhibited a pronounced cytotoxic effect when combined with Cu(II) (Figure 3C). Furthermore, the CEL+NIR combination therapy significantly enhanced cytotoxicity compared to the CL+NIR treatment (Figure 3D). These results indicate that CEL NP not only exhibit excellent biocompatibility but also effectively induce tumor cell cytotoxicity in response to photothermal stimulation.

Furthermore, the antitumor activity of CEL NP was assessed using a calcein‐AM and propidine iodide (PI) live/dead staining assay. Fluorescence images revealed that the highest number of death cells (red) was observed in CT26 cells treated with CEL+NIR, indicating strong tumor growth inhibition through the combined effects of amplified cuproptosis and PTT/CDT. In contrast, a considerable number of live cells (green) were present in the CL+NIR and Cu_2−_ *_X_* S treatment groups, suggesting that while PTT/CDT and CDT showed significant tumor cell suppression, they did not achieve optimal antitumor efficacy. Negligible red fluorescence signals were detected in the PBS, NIR, CL, and CEL groups, confirming the biosafety of both laser irradiation and the drugs when protected by the LA coating (Figure 3G,J). The percentage of annexin V (AV) positive cells under the aforementioned treatments was assessed. Quantitative FCM analysis indicated that the AV‐positive rate of CT26 cells in the CEL+NIR subgroup reached 65.50%, which was 1.39 times higher than that in the CL+NIR treatment group (47.07%) and 2.40 times higher than that in the Cu_2−_ *_X_* S treatment group (27.24%). In contrast, only ≈7%–13% of the AV‐positive cells were detected in the remaining groups (Figure 3H,K). Collectively, these findings suggest that CEL NP, when activated by a 1064 nm laser, can precisely induce a tumor‐killing effect on CT26 cells in vitro.

To assess the ROS generation of CEL NP in vitro, intracellular ·OH levels in CT26 cells were detected using the fluorescent probe dichlorodihydrofluorescein diacetate (DCFH‐DA).^[^ ^40^ ^]^ As shown in Figure 3E, fluorescence images revealed no significant fluorescence signal in the groups of PBS, NIR, CL, and CEL. However, a progressive increase in fluorescence intensity was observed in the Cu_2−_ *_X_* S, CL+NIR, and CEL+NIR treatment groups, indicating that Cu(I) ions induced intracellular ROS production via a Fenton‐like reaction. Among these, CEL+NIR treatment displayed the most intense DCFH fluorescence, likely due to ES, traditionally considered an oxidative stress inducer, further amplifying ROS production (Figure 3I).^[^ ^41^ ^]^ Consistently, FCM analysis quantitatively confirmed that ·OH levels were highest in CT26 cells treated with CEL+NIR, showing as 1.56‐fold increase compared to the CL+NIR treatment group and 2.96‐fold increase compared to the Cu_2−_ *_X_* S treatment group (Figure 3F). These results demonstrate that CEL NP significantly enhance ROS generation through self‐amplified CDT.

#### 2.2.2 In Vitro Analysis of Cuproptosis‐Related Pathways and ICD Induction

**Figure 4:**
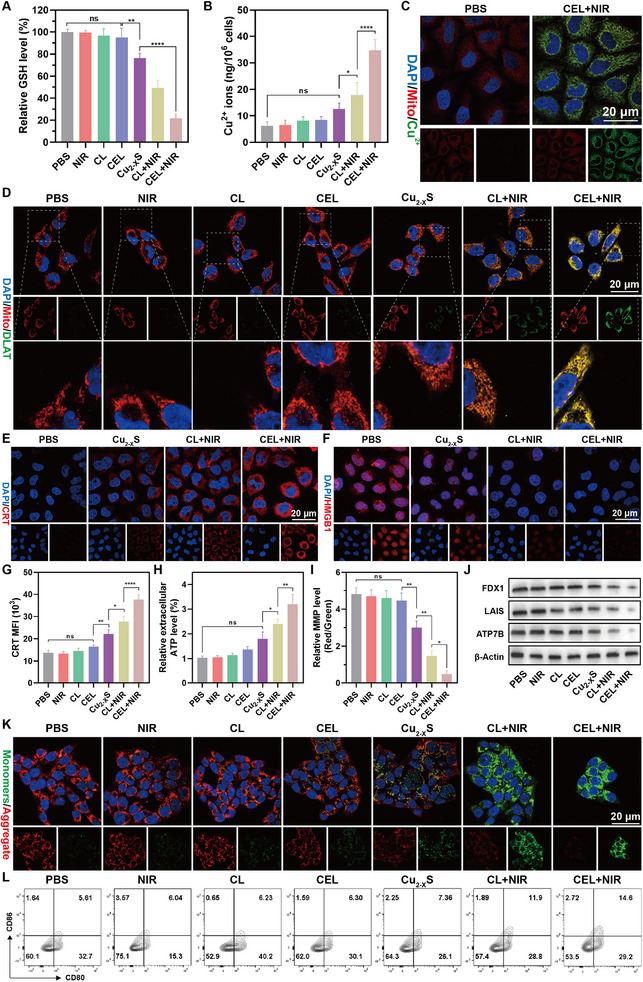
A) Intracellular relative GSH levels and B) copper ions concentration in CT26 cells after different treatments (*n* = 5). C) CLSM images of the Cu^2+^ probe in CT26 cells after PBS and CEL+NIR treatments. D) CLSM images displaying the aggregation of DLAT protein in CT26 cells after various treatments. CLSM images of E) CRT and F) HMGB1 in CT26 cells after major treatments. G) Quantification of CRT exposure after various treatments (*n* = 3). H) Relative extracellular ATP levels in the supernatant of CT26 cells after various treatments (*n* = 5). I) Quantitative analysis of relative MMP levels through the ratio of red/green fluorescence intensity in CT26 cells after various treatments (*n* = 3). J) Western blotting analysis of FDX1, LIAS, and ATP7B protein expression in response to different treatments. K) Fluorescence images of CT26 cells stained with JC‐1 indicating changes in mitochondrial membrane potentials (MMP) after various treatments. L) FCM data of the proportion of matured BMDCs after various treatments. Data are presented as mean ± SD. *p*‐values were calculated by one‐way ANOVA test. *p < 0.05, **p < 0.01, ***p < 0.001, ****p < 0.0001, ns, not significant.

The depletion of intracellular GSH has the potential to sensitize ROS‐based therapy and promote cuproptosis.^[^ ^42^ ^]^ Intracellular GSH levels were measured using a GSH content assay kit. In this assay, DTNB is reduced by GSH to yield TNB, a yellow compound with a maximum absorbance at 412 nm. Given the excellent linear relationship between the absorbance peak and the reduced GSH concentration, intracellular GSH levels were determined by measuring the change in the absorption peak before and after the reaction (Figure S9, Supporting Information). As shown in **Figure** **4**A, following the treatments with Cu_2−_ *_X_* S, CL+NIR, and CEL+NIR, intracellular GSH levels decreased by 23.60%, 50.98%, and 78.43%, respectively. This reduction can be attributed to the oxidative properties of Cu(II) released from the Cu_2−_ *_X_* S HNSs.^[^ ^43^ ^]^

Accordingly, intracellular copper ion analysis showed elevated copper levels in the CL+NIR group, likely due to the increased Ksp of the Cu_2−_ *_X_* S solution following irradiation. This elevation was even more pronounced in the CEL+NIR group, which can be attributed to the chelating function of ES, facilitating copper ion accumulation within tumor cells (Figure 4B). Notably, ES not only facilitated intracellular copper ion accumulation within tumor cells by chelation but also inhibited its efflux by degrading copper‐transporting ATPases.^[^ ^44^ ^]^ ATP7B, a key member of the copper‐transporting ATPase family, facilitates copper efflux through ATP consumption.^[^ ^45^ ^]^ Western blot analysis demonstrated ATP7B degradation in CT26 cells following CEL+NIR treatment (Figure 4J). These results indicate that CEL+NIR treatment disrupts intracellular copper homeostasis through a dual mechanism of “open source and reduce expenditure.”

Considering the copper‐chelating ability of CEL NP, the induction of the cuproptosis pathway was further evaluated. Intracellular Cu(II) can accumulate in mitochondria and bind to DLAT, leading to DLAT aggregation and destabilization of iron‐sulfur (Fe‐S) cluster proteins, including ferredoxin (FDX1) and lipoyl synthase (LIAS).^[^ ^46^ ^]^ Immunofluorescence images were obtained to visualize the aggregation of DLAT. As shown in Figure 4D, the CEL+NIR group displayed the most pronounced DLAT foci. In addition, western blot analysis was performed to assess the levels of FDX1 and LAIS. The results indicated that CEL+NIR treatment led to a significant downregulation of intracellular FDX1 and LAIS expression (Figure 4J). Considering the differences in the expression of hallmark proteins associated with cuproptosis, these findings confirm that CEL NP can effectively trigger cuproptosis in response to photothermal stimulation.

Given that mitochondria are the primary target organelles for cuproptosis, mitochondrial copper overload is a critical prerequisite for inducing this cell death mechanism.^[^ ^47^ ^]^ Colocalization immunofluorescence imaging of copper ions and Mito‐tracker in CT26 cells confirmed substantial copper accumulation in mitochondria following CEL+NIR treatment, with significant overlap between green copper signals and red mitochondrial fluorescence (Figure 4C; Figure S10, Supporting Information). To further assess mitochondrial function, we evaluated the mitochondrial membrane potential (MMP) of CT26 cells after various treatments using the fluorescent probe JC‐1 to monitor MMP levels. JC‐1 forms red‐fluorescing polymers in healthy mitochondria with high MMP, while it forms green‐fluorescing monomers in damaged mitochondria, signaling depolarization. CLSM images revealed that CEL+NIR treatment induced a pronounced green fluorescence signal, indicative of severe mitochondrial damage, with the red‐to‐green fluorescence ratio being significantly lower in the CEL+NIR group compared to other treatment (Figure 4K,I). In addition, mitochondrial permeability transition pore (mPTP) is a key indicator of mitochondrial function, which led us to evaluate the opening of mPTP in mitochondria after various treatments. FCM data indicated increased mPTP opening, correlating with a decrease in FITC fluorescence, signifying mitochondrial stress following CEL+NIR treatment (Figure S11, Supporting Information). Collectively, these results confirm that CEL+NIR treatment induces cuproptosis and severely compromises mitochondrial function in tumor cells.

ICD is a regulated form of cell death triggered by the release of DAMPs from dying cancer cells, leading to an enhanced adaptive immune response. A recent study has suggested that protein aggregation in mitochondria caused by cuproptosis can promote proteotoxic stress, ultimately activating the antitumor immune response via ICD.^[^ ^48^ ^]^ Therefore, we evaluated the connection between CEL+NIR‐induced cuproptosis and the ICD pathway. The translocation of CRT across the cytomembrane, a hallmark of ICD, was visualized using CLSM. Cells treated with CL+NR exhibited strong CRT fluorescence signals on the surface of cells, whereas CEL+NIR treatment further enhanced CRT expression, with a fluorescence intensity 1.46 times higher than CL+NIR treatment, indicating that cuproptosis can further enhance CRT exposure compared with PTT/CDT alone (Figure 4E,G; Figure S12A, Supporting Information). Next, we investigated the secretion of HMGB1 from the nucleus. CLSM images showed strong HMGB1 fluorescence signals overlapping with DAPI staining in cells treated with PBS, NIR, CL, and CEL, while varying degrees of HMGB1 migration were observed in cells treated with CL+NIR and CEL+NIR (Figure 4F; Figure S12B,C, Supporting Information). In addition, we quantified the extracellular release of ATP following various treatments using an ATP assay kit. The highest ATP release was detected in the supernatant of cells treated with CEL+NIR, which was 3.04 and 1.36 times higher than that of PBS and CL+NIR, respectively (Figure 4H). Recent research suggests that a reduction of intracellular ATP can affect the expression and function of Cu‐ATPases (ATP7A/B), further inhibiting copper ion efflux and reinforcing the cuproptosis effect.^[^ ^45^ ^]^ Numerous studies have demonstrated that DAMP signals trigger the maturation of DCs, subsequently enhancing specific T lymphocyte‐mediated antitumor responses.^[^ ^49^ ^]^ Therefore, the maturation of bone‐marrow‐derived DCs (BMDCs) was evaluated after coculturing them with CT26 cells under various treatment conditions. CT26 cells treated with CEL+NIR, when cocultured with BMDCs, resulted in a significant increase in mature BMDCs to 14.50%, which was 2.46, 1.90, and 1.32 times higher than with PBS, Cu_2−_ *_X_* S, and CL+NIR treatments, respectively (Figure 4L; Figure S13, Supporting Information). DCs can secrete cytokines, including IFN‐γ and TNF‐α, which can modulate the intensity and duration of immune responses.^[^ ^50^ ^]^ Following different treatments, cytokines, including TNF‐α and IFN‐γ, were measured in the supernatant of DCs, and CEL+NIR demonstrated the highest cytokine secretion (Figure S14, Supporting Information). Overall, these findings demonstrate that CEL+NIR‐induced cuproptosis can effectively trigger ICD and reverse the immunosuppressive TME.

### 2.3 In Vivo Synergistic Antitumor Capacity and Immune Activation of CEL NP

**Figure 5:**
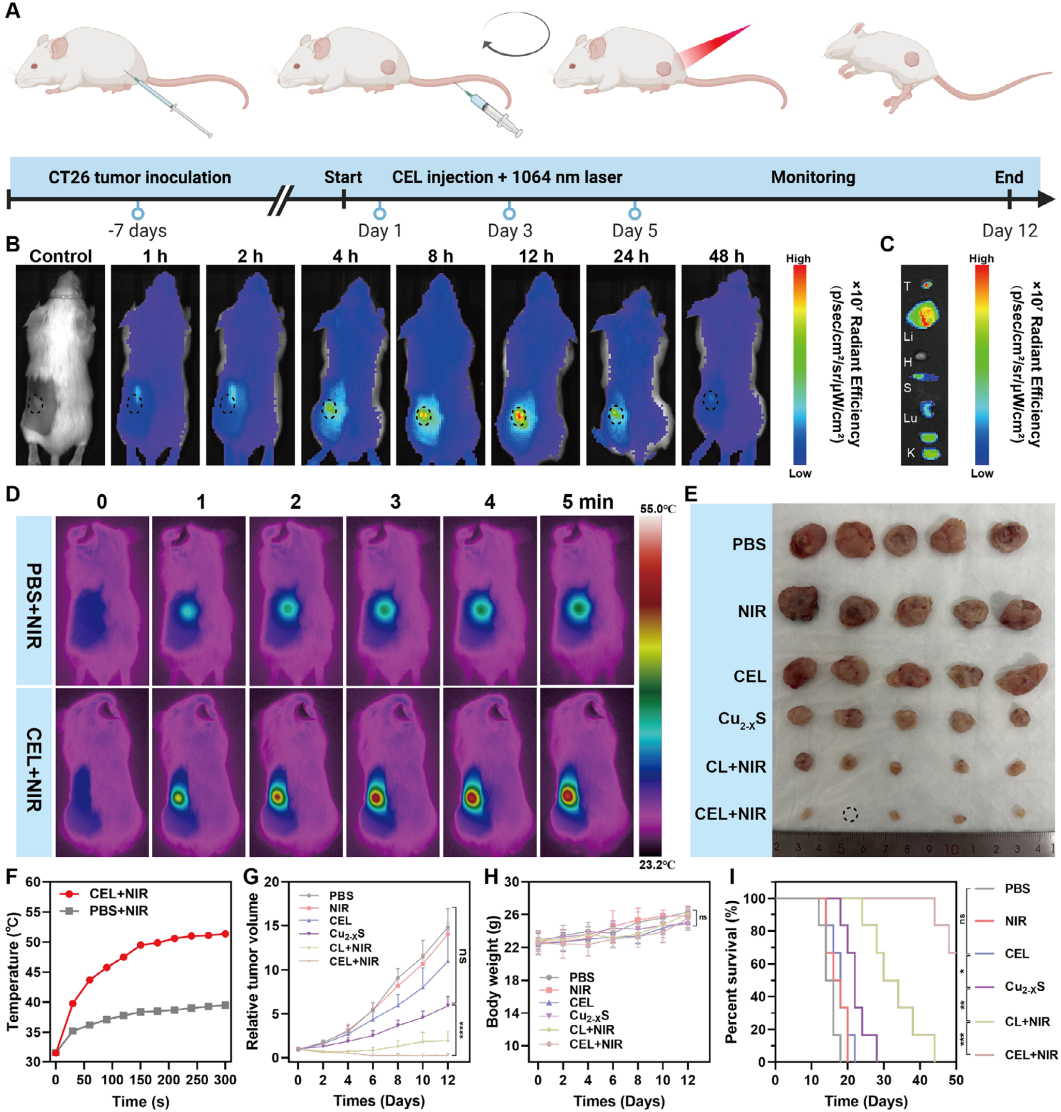
In vivo synergistic amplified cuproptosis combining PTT/CDT anticancer effect in CT26 tumor‐bearing mice. A) Schematic of the experimental schedule for CT26 tumor‐bearing mice (Schematics were created with BioRender.com). B) Biodistribution in vivo after intravenous injection of CEL‐ICG NP at different time points. C) Ex vivo imaging of CEL‐ICG NP in the tumor and major organs (Li: liver; H: heart; S: spleen; Lu: lung; K: kidneys). D) Photothermal images and F) corresponding temperature curves of CEL+NIR or PBS+NIR (1064 nm, 1 W cm^−2^, for 5 min). E) Photographs of the harvested tumors. G) Tumor growth curves during the treatment period (*n* = 5). H) Body weight of CT26 tumor‐bearing mice in the different groups (*n* = 5). I) Survival rates documented from the start of treatments up to the period of 50 days. Survival analysis was conducted using the Kaplan‒Meier method, with statistical significance assessed via the log‐rank test. Data are presented as mean ± SD. *p*‐values were calculated by one‐way ANOVA test. *p < 0.05, **p < 0.01, ***p < 0.001, ****p < 0.0001, ns, not significant.

To evaluate the therapeutic efficiency of CEL NP in vivo, a subcutaneous CT26 tumor cell model was established. The experimental design is illustrated in **Figure** **5**A. The biodistribution and tumor accumulation were assessed using ICG‐labeled CEL NP. The CEL‐ICG NPs were injected via the tail vein into CT26 tumor‐bearing mice, followed by the observation of fluorescence intensity using an In Vivo Imaging System (IVIS). As shown in Figure 5B, fluorescence signals were initially localized in the liver and spleen at 2 h post‐injection. Over time, the fluorescence intensity at the tumor site increased, peaking at 12 h, with significant accumulation still evident at 24 hours. Subsequently, the tumors and major organs (liver, kidney, heart, lung, and spleen) were harvested for ex vivo biodistribution analysis at the 12‐h mark. Imaging results revealed that fluorescence intensity at the tumor site remained notably elevated, highlighting effective targeting ability of CEL‐ICG NP (Figure 5C). Collectively, these results confirm the tumor accumulation capability of CEL NP and suggest that an optimal time window for photothermal treatment (PTT) occurred ≈12 h following intravenous administration.

Next, the in vivo photothermal performance of CEL NP was assessed. CT26 tumor‐bearing mice, injected with either PBS or CEL NP, were subjected to NIR‐II irradiation (1064 nm, 1 W cm^−2^, for 5 min) 12 h post‐injection to examine the photothermal effect. The temperature at the tumor site was monitored using an infrared thermal camera (Fortric 600c). The results indicated that the tumor site temperature escalated to 51.4 °C (Δ*T* = 19.9 °C) in the CEL NP group, while it only reached 39.5 °C (Δ*T* = 7.9 °C) in the PBS control group (Figure 5D,F). This confirmed that CEL NP also exhibit robust photothermal properties in vivo. Subsequently, we further evaluated the enhanced cuproptosis combined with PTT/CDT on the antitumor effects of CEL NP in vivo. The CT26 tumor‐bearing mice were randomly divided into six treatment groups: (1) PBS, (2) NIR, (3) CEL, (4) Cu_2−_ *_X_* S, (5) CL + NIR, and (6) CEL+NIR. NIR‐II irradiation (1064 nm, 1 W cm^−2^, for 5 min) was performed 12 h after the nanoparticles were administered via the tail vein. At the end of the treatment, all tumors were excised and photographed (Figure 5E). The relative tumor volumes in the PBS group increased to ≈12 times their initial size over 12 d. The CEL NP group showed no significant tumor inhibitory effect due to the protective LA coating. Treatment with Cu_2−_ *_X_* S HNSs led to a reduction in relative tumor volume due to CDT, but this was limited by insufficient ROS generation. In contrast, the CL+NIR subgroup exhibited enhanced antitumor effects due to improved PTT/CDT synergy. Notably, the combined therapy using CEL+NIR achieved the most significant tumor suppression compared to other groups, which was attributed to the synergistic effects of cuproptosis and PTT/CDT (Figure 5G). Moreover, the CEL+NIR treatment significantly improved the survival rates of CT26 tumor‐bearing mice compared to the other treatment groups (Figure 5I). In addition, over the treatment period, there were no significant differences in body weight across the groups, indicating that CEL NP possesses excellent biocompatibility in vivo (Figure 5H).

**Figure 6:**
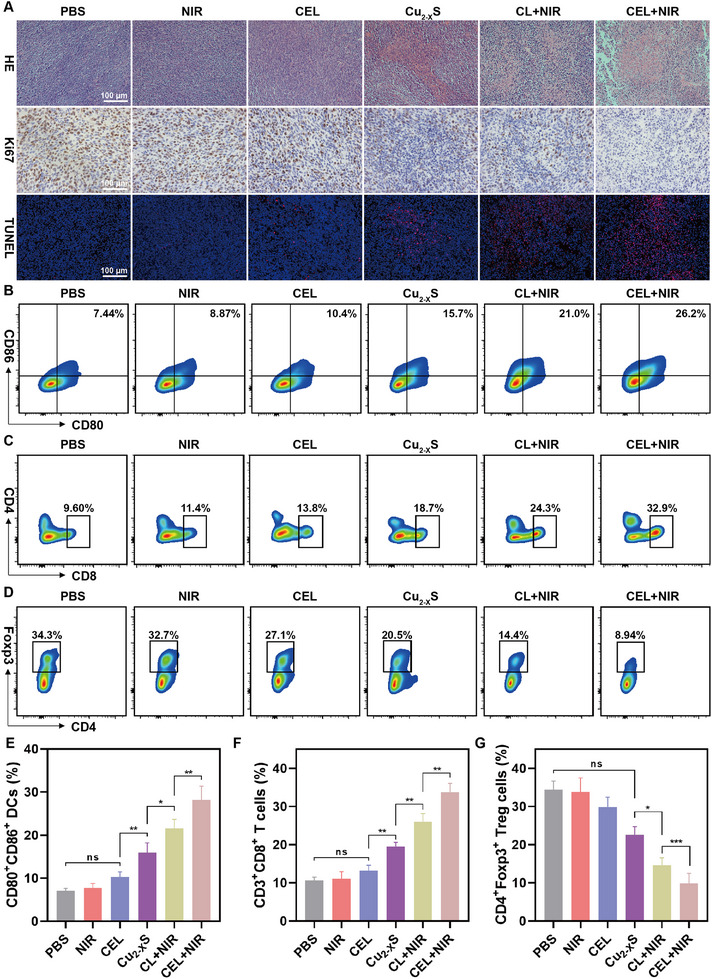
A) H&E‐, KI67‐, and TUNEL‐stained tumor tissues collected from different groups at the end of the various treatments. B) Representative flow cytometry analysis and E) corresponding statistical comparisons of DCs (CD80^+^, CD86^+^) maturation from tumor‐draining lymph nodes after various treatments. C) Representative flow cytometry analysis and F) corresponding statistical comparisons of CD8^+^ T cells in primary tumor tissue after various treatments. D) Representative flow cytometry analysis and G) related quantification of Tregs (Foxp3^+^, CD4^+^) infiltration in CT26 tumors after various treatments (*n* = 4). Data are presented as mean ± SD. *p*‐values were calculated by one‐way ANOVA test. *p < 0.05, **p < 0.01, ***p < 0.001, ****p < 0.0001, ns, not significant.

Furthermore, the apoptotic status and proliferative activity of tumor samples in each treatment group were examined. Hematoxylin and eosin (H&E), Ki67 Immunohistochemistry (IHC), and TUNEL staining method provided the pathological evidence to support the antitumor efficacy across all groups. H&E staining revealed that synergistic treatment with CEL+NIR resulted in the highest necrosis rate among all groups analyzed (**Figure** **6**A). Chromosomal DNA breaks, indicative of late apoptosis, were detected using dUTP‐labeled TUNEL. The greatest number of TUNEL‐positive cells was observed in the CEL+NIR group (Figure 6A). In addition, Ki67 expression was significantly inhibited after CEL+NIR treatment, indicating that the proliferative ability of the tumor cells was severely impaired (Figure 6A; Figure S15, Supporting Information).

To demonstrate the activation of the immune system mediated by CEL+NIR treatment, tumor samples and lymph node tissues from treated mice were collected, and the proportions of immune cells were quantified by FCM. The ICD effect triggered by augmented cuproptosis facilitated the infiltration of tumor‐related immune cells.^[^ ^51^ ^]^ The results indicated that the maturation of DCs (CD80^+^CD86^+^) increased to 28.2%, which was 4.02 and 1.32 times higher than those treated with PBS and CL+NIR, respectively (Figure 6B,E). Mature DCs present antigens to T cells, thereby activating T cell infiltration and reversing immunosuppression within the TME.^[^ ^52^ ^]^ Accordingly, a significant increase in CD3^+^CD8^+^ T cell (33.7%) infiltration within tumors was observed in the CEL+NIR treatment group, which was significantly higher than the other treatment subgroups (Figure 6C,F). Conversely, the proportion of Tregs (CD4^+^FoxP3^+^) decreased to 9.85% after CEL+NIR treatment, further alleviating the immune tolerance of CD8+ cytotoxic T lymphocytes (CTLs) toward cancer cells (Figure 6D,G). These results confirm that cuproptosis combined with PTT/CDT triggers ICD and activate a robust immune response.

To further evaluate the biosafety of CEL NP, the biochemical indices of mice subjected to different treatment protocols were evaluated. Hepatic function tests, including alanine aminotransferase (ALT) and aspartate aminotransferase (AST), along with renal function assessments such as creatinine (CREA) and blood urea nitrogen (BUN), remained within normal ranges, with no significant differences observed among the groups (Figure S16, Supporting Information). Moreover, the pathology of the major organs following different treatments was also examined. H&E staining revealed no histological alterations or tissue damage in the liver, heart, spleen, lungs, and kidneys compared with the PBS control group (Figure S17, Supporting Information). Based on these results, enhanced cuproptosis when combined with PTT/CDT of CEL+NIR treatment achieved the highest tumor suppression rate while maintaining excellent biocompatibility in vivo.

## 3 Conclusions

In this study, we developed CEL NP by encapsulating ES within Cu_2−_ *_X_* S HNSs, leveraging photothermal energy for cuproptosis induction. Upon NIR‐II irradiation, the phase‐change material LA in CEL NP melts, facilitating the controlled release of both ES and Cu(II) within tumor cells. This design overcomes the pharmacokinetic challenges associated with ES, which has a short half‐life in the bloodstream and rapid metabolism. ES then aids in the mitochondrial uptake of Cu(II), depleting GSH and generating Cu(I), which promotes DLAT aggregation and reduces Fe‐S cluster proteins, regulated by FDX1. The toxic aggregation of cuproptosis‐related proteins increases exposure to DAMPs, which reshape the immunosuppressive TME by triggering ICD. CEL NP demonstrates potent anticancer effects through this “copper‐dependent cell death” mechanism, both in vitro and in vivo, while maintaining low toxicity to normal tissues. Our findings suggest that CEL NP represents a novel strategy for CRC treatment by enhancing immunotherapy efficacy through precisely targeted cuproptosis under photothermal conditions.

## Conflict of Interest

The authors declare no conflict of interest.

## Supporting information



Supporting Information

## Data Availability

The data that support the findings of this study are available on request from the corresponding author. The data are not publicly available due to privacy or ethical restrictions.
