# Supplementary material for: A Copper‐Based Photothermal‐Responsive Nanoplatform Reprograms Tumor Immunogenicity via Self‐Amplified Cuproptosis for Synergistic Cancer Therapy
Source: Adv Sci (Weinh). 2025 Mar 24;12(19):2500652. doi: 10.1002/advs.202500652 (PMC12097029; doi:10.1002/advs.202500652)
Supplement: Supplementary file 1 — Supporting Information [file ADVS-12-2500652-s001.docx]

**Supplementary Information (SI)**

**A Copper Based Photothermal-Responsive Nanoplatform Reprograms Tumor Immunogenicity via Self-Amplified Cuproptosis for Synergistic Cancer Therapy**

Runzi Cheng ^#, a, b^, Zhenhao Li ^#, a, c^, Weican Luo ^#, a, b^, Hongwu Chen ^#, d^, Tingting Deng ^b, e^, Zhenqi Gong ^a, b^, Qing Zheng ^a, b^, Baizhi Li ^a, b^, Yongming Zeng ^a,^ *, Huaiming Wang ^a,^ *, Cong Huang ^e,^ *

1. Department of Gastrointestinal Surgery, The First Affiliated Hospita l of Shantou University Medical College, Shantou, 515041, China.
2. Shantou University Medical College, Shantou, 515041, China.
3. Southern Medical University, Guangzhou, 510515, China
4. Department of Neurosurgery, The First Affiliated Hospital of Shantou University Medical College, Shantou, 515041, China.
5. Department of Ultrasound, The First Affiliated Hospital of Shantou University Medical College, Shantou 515041, China.

#. Contributed equally.

*Corresponding authors.

E-mail address:

Yongming Zeng ymzeng1@stu.edu.cn;

Huaiming Wang [17f1hmwang@stu.edu.cn](mailto:17f1hmwang@stu.edu.cn);

Cong Huang 08chuang@stu.edu.cn.

**Materials and methods**

**Materials**

All chemicals used were of analytical grade and were used as received without further purification. Copper chloride dihydrate (CuCl_2_·2H_2_O), polyvinylpyrrolidone (PVP K30, MW = 40 000 Da), sodium hydroxide (NaOH), ascorbic acid (AA), and sodium sulfide nonahydrate (Na_2_S·9H_2_O) were purchased from Shanghai Aladdin Biochemical Technology Co., Ltd., Elesclomol (ES) and lauric acid (LA) were purchased from MedChemExpress Co., Ltd., Deionized water (DI water, 18.2 MΩ cm) produced by a Milli-Q water system was used for all the experiments.

**Synthesis of** **Cu_2-X_S hollow nanospheres (HNSs)**

Cu_2_O nanospheres (NSs) were synthesized by adding AA to aqueous solutions of Cu(Ⅱ) and NaOH, using PVP as a capping agent. Specifically, 0.6 g of PVP and 0.5 mmol of CuCl_2_·2H_2_O were dissolved in 20 mL of DI water, and then 0.32 g of NaOH aqueous solution was added. After magnetic stirring for 5 min, 1.0 mmol of AA was added to the above mixture and stirred magnetically for an additional 5 min. The addition of AA immediately resulted in the formation of orange Cu_2_O NSs. The Cu_2_O NSs were collected by centrifugation at 6000 rpm for 5 min, and thoroughly washed several times with DI water and ethanol. All experiments were conducted at room temperature. Subsequently, Cu_2-X_S HNSs were obtained from the Cu_2_O NSs suspension used as templates, by adding 0.125 mmol of Na_2_S⋅9H_2_O solution, followed by magnetic stirring for 1.5 h. The samples were washed with DI water and ethanol through centrifugation. The final samples were collected by centrifugation, washed repeatedly with DI water and ethanol, and then dried by vacuum lyophilization before being stored at 4℃ for further use. The synthesis process could be explained by the relevant chemical equation.

CuCl_2_ + NaOH → Cu(OH)_2_ + NaCl

Cu(OH)_2_ + AA (C_6_H_8_O_6_) → Cu_2_O + C_6_H_6_O_6_

Cu_2_O + S^2-^ +H_2_O → Cu_2-X_S + OH^-^

**Synthesis of CEL NP**

A 5 mg/mL suspension of Cu_2-X_S HNSs was ultrasonically dispersed and mixed with an ES solution to achieve a final concentration of 10mM. The Cu_2-X_S@ES (CE) nanoparticles were obtained and collected after overnight magnetic stirring, followed by centrifugation with DI water. For LA modification, the CE nanoparticles were suspended in 10mL of PBS buffer, to which 10mg of LA was added and ultrasonically dispersed to ensure the melting of LA. The mixture was vigorously stirred at 47℃ for 12 h. The CEL NP system was obtained after centrifugation at 8000 rpm for 10 min, and washed for 3 times with DI water. The final product was dried by vacuum lyophilization and stored at 4℃ for further use. The Cu_2-X_S@LA (CL) nanoparticles were prepared in a similar manner, but without the addition of ES.

**Characterization of CEL NP**

The crystal structure and morphology of the products were characterized using power X-ray diffraction (XRD, Rigaku ULTIMA IV), X-ray photoelectron spectroscopy (XPS, Thermo Scientific K-Alpha) and field emission scanning electron microscopy (FESEM, Zeiss Sigma 300). Transmission electron microscopy (TEM) and element mapping were performed using high-resolution transmission electron microscopy (HR-S/TEM, Thermo Scientific Talos F200iS/TEM). The assembly of LA was assessed by differential scanning calorimetry (DSC, Mettler TGA/DSC3+). The UV-vis/NIR spectra of the samples were measured using a spectrophotometer (UV-250 IPC). The dynamic light scattering (DLS) size distribution and zeta potential of the samples were determined using a Zetasizer Nano AS.

**Calculation of ES loading capacity**

Standard ES solutions with concentrations of 1.5, 3, 6 and 12 mM were prepared, and their UV-vis spectra were measured using a spectrophotometer. A standard curve of ES concentration was then generated based on the relationship of the UV-vis spectra peaks and the standard concentrations. 5 mg of Cu_2-X_S HNSs were dispersed in 1 mL of 5 mM ES solution and stirred overnight at room temperature. The excess ES was removed by centrifugation and washing with DI water. The drug loading capacity of ES was calculated by comparing the absorbance difference between the supernatant and the original ES solution. The max ES loading rate and the drug loading capacity in subsequent in vitro experiments are calculated using the following formula.

ES loading rate = (loaded ES) / (Cu_2-X_S+ loaded ES) × 100%.

**NIR-Ⅱ photothermal performance of Cu_2-X_S HNSs**

To evaluate the photothermal performance of the nanoparticles, Cu_2-X_S HNSs solutions with different concentrations (0-200 μg/mL) were continuously irradiated with a NIR-Ⅱ region (1064 nm) laser (Changchun New Industries Optoelectronics Tech. Co., Ltd., China) at various power densities (0.7, 1, 1.5 W/cm^2^) for 5 minutes. The temperature of the solution was monitored and recorded using an infrared thermal camera (Fortric 600c, FOTRIC Thermal Intelligence Co., Ltd., China). Subsequently, a 50 μg/mL solution of Cu_2-X_S was irradiated (1064 nm, 1 W/cm^2^, for 5 min) until the temperature stabilized. The laser was then turned off, allowing the solution to cool naturally to room temperature. The photothermal conversion efficiency (*η*) of the Cu_2-X_S HNSs was calculated according to the methodology described in the previously published literature [1]. In addition, four laser on/off cycles were performed to evaluate the photothermal stability of the Cu_2-X_S HNSs. The *η* of Cu_2-X_S was calculated according to the following equations.


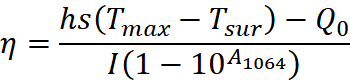


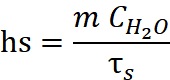


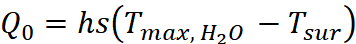


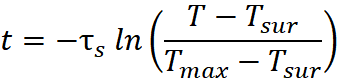


where *T*_max_ refers to the highest temperature of the solution, *T*_surr_ is the ambient temperature, *I* is the laser power, *A_1064_* is the absorbance of Cu_2-X_S@Elesclomol@LA at 1064 nm, *m* is the mass of the solution, and *C_H2O_* is the specific heat capacity of water.

**Photothermal-response release capacity of Cu(Ⅱ) and ES from CEL NP**

A 200 μg/mL solution of CEL NP was prepared for the drug release experiment. One sample was irradiated (1064 nm, 1 W/cm^2^, for 5 min), while the other samples remained untreated for 7 hours. At different time points, all samples were centrifuged at 8000 rpm for 10 minutes, and the supernatant was collected. The content of Cu(Ⅱ) released was measured using inductively coupled plasma mass spectrometry (ICP-MS, Agilent 7700). The concentration of released ES was calculated based on the standard curve generated from the UV-vis spectra peak at 380 nm.

**Hydroxyl free radical (•OH) generation of Cu_2-X_S HNSs**

To analyze the production of •OH by Cu_2-X_S HNSs, we employed a classic colorimetric method based on the degradation of methylene blue (MB) by •OH. The absorbance at 664 nm of MB (20 μg/mL) in phosphate-buffered saline (PBS; pH 7.0) with varying concentrations (0-0.72 mM) of H_2_O_2_ was examined after the addition of Cu_2-X_S HNSs (40 μg/mL). Additionally, the reaction temperature was raised to 47℃ to simulate the heat generated during PTT, and the PH was adjusted to 6.0 to simulate the slightly acidic TME. The absorbance was also assessed to detect the generation of •OH under these conditions. To avoid any potential interference from the Cu_2-X_S HNSs in the absorbance measurements, the samples were centrifuged for 5 min prior to measurement.

**Cellular uptake analysis**

 The CT26 cells were incubated in Roswell Park Memorial Institute (RPMI) 1640 medium supplemented with 10% fetal bovine serum (FBS) and 1% Penicillin-Streptomycin. The cells were cultured in a dark incubator at 37 ℃ with 5% CO_2_. First, CT26 cells (3×10^5^ cells/well) were seeded in 6-well plates and cultured for 24 hours. Subsequently, the cells were incubated with rhodamine B-labelled CEL NP (CEL-RB NP) for various time periods. The cells were then fixed with 4% paraformaldehyde, and the cell nuclei were stained with DAPI. Images of intracellular uptake were collected using a confocal laser scanning microscopy (Zeiss LSM800). Finally, intracellular fluorescence was quantified using flow cytometry (FCM; BD Accuri C6).

**In vitro photothermal effect of CL NP**

CT26 cells (8×10^3^ cells/well) were seeded in 96-well plates and cultured for 24 hours. The cells were then incubated with different concentrations of CL (0-200 μg/mL) in 200 μL of complete medium. After washing the cells with PBS three times, they were illuminated with a 1064 nm laser (1 W/cm^2^) for 5 and 8 minutes. Finally, cell viabilities were assessed using the Cell Counting Kit-8 (CCK-8) assay.

**In vitro cytotoxicity evaluation**

Human umbilical vein endothelial cells (HUVECs) and CT26 cells (8×10^3^ cells/well) were incubated in 96-well plates for 24 hours. The cells were then incubated with various concentrations of Cu_2-X_S (0-200 μg/mL), CEL (0-200 μg/mL), ES (0-200 nM), and ES (0-200 nM) + 1 μM CuCl_2_ for 24 hours. Cell variability was evaluated using the standard CCK-8 assay. In addition, the viability of CT26 cells was measured under the following treatment conditions: PBS, NIR, CL, CEL, Cu_2-X_S, CL+NIR, and CEL+NIR ([Cu]: 25μg/mL, NIR: 1064 nm, 1 W/cm^2^, for 8 min).

**In vitro live-dead cell staining experiment**

The live-dead cell status was visualized using a Calcein-AM/PI double staining kit (Beijing Solarbio Science & Technology Co., Ltd., China). CT26 cells (1.5×10^5^ cells/well) were seeded in 12-well plates and incubated for 24 hours, followed by treatment with PBS, NIR, CL, CEL, Cu_2-X_S, CL+NIR, CEL+NIR ([Cu]: 25μg/mL, NIR: 1064 nm, 1 W/cm^2^, for 8 min) for another 24 hours. Finally, all cells were harvested, collected, and co-stained with the Calcein-AM/PI double stain kit. CLSM was used to capture the images.

**Cellular apoptosis analysis**

The In vitro cell apoptosis rate was examined using an Annexin V-FITC apoptosis detection kit (Shanghai Yeasen Biotechnology Co., Ltd., China). Briefly, CT26 cells were seeded in 12-well plates at a density of 1.5×10^5^ cells/well and incubated for 24 hours. The CT26 cells were then treated with PBS, NIR, CL, CEL, Cu_2-X_S, CL+NIR, and CEL+NIR ([Cu]: 15μg/mL, NIR: 1064 nm, 1 W/cm^2^, for 8 min) for another 24 hours. After washing the cells with PBS three times, the CT26 cells were harvested by trypsinization without EDTA and co-incubated with the Annexin reagent (protected from light) for 15 minutes, followed by the PI reagent for 5 minutes at room temperature. The collected cells were then washed with PBS and measured by FCM within 1 hour.

**In vitro detection of reactive oxygen species (ROS) production**

The generation of ROS was evaluated using a reactive oxygen species assay kit (Beijing Solarbio Science & Technology Co., Ltd., China). CT26 cells (1.5×10^5^ cells/well) were seeded into 12-well plates and incubated for 24 hours. Subsequently, the CT26 cells were subjected to various treatments: PBS, NIR, CL, CEL, Cu_2-X_S, CL+NIR, and CEL+NIR ([Cu]: 15μg/mL, NIR: 1064 nm, 1 W/cm^2^, for 8 min) for 8 hours. After washing the cells with PBS three times, the CT26 cells were co-incubated with a serum-free medium containing the ROS indicator dichlorodihydrofluorescein diacetate (DCFH-DA, 10 μM) for 30 minutes. Finally, the intracellular ROS status was detected using fluorescence microscope and FCM analysis.

**In vitro glutathione (GSH) depletion experiment**

The depletion of GSH in CT26 cells was assessed using a reduced GSH content assay kit (Beijing Solarbio Science & Technology Co., Ltd., China). CT26 cells were seeded and incubated for 48 hours until they reached a density of 10^6^ cells/well. After washing the cells with PBS three times, the CT26 cells were treated with PBS, NIR, CL, CEL, Cu_2-X_S, CL+NIR, and CEL+NIR ([Cu]: 15μg/mL, NIR: 1064 nm, 1 W/cm^2^, for 8 min) for 2 hours. After treatment, the CT26 cells were collected and broken by repeated freeze-thaw cycles 2-3 times (frozen in liquid nitrogen and dissolved in a 37℃-water bath). The resulting supernatant was collected by centrifugation and placed on ice for measurement using a microplate reader. The relative GSH levels were assessed according to the manufacturer’s instructions.

**In vitro intracellular copper ion accumulation experiment**

CT26 cells were seeded in 6-well plates and cultured for 24 h. The cells were then treated with PBS, NIR, CL, CEL, Cu_2-X_S, CL+NIR, and CEL+NIR ([Cu]: 15μg/mL, NIR: 1064 nm, 1 W/cm^2^, for 8 min). Following treatment, the cells were incubated at 37°C for 2 h. The cells were then washed three times with PBS. The CT26 cells were further processed by co-incubation with a FITC-labeled copper ion probe to detect copper accumulation. The localization of copper ions within the mitochondria was assessed using CLSM, with co-staining using a Mitotracker dye.

Following similar treatment protocols, the cells were collected, washed, and then digested with nitric acid. The copper ion concentration was subsequently measured using Atomic Absorption Spectroscopy (AAS). To evaluate Cu-ATPases, the treated cells were washed three times with PBS for western blotting to detect the protein expression of ATP7B.

**In vitro evaluation of mitochondrial damage due to cuproptosis**

CT26 cells were seeded into 6-well plates at a density of 3×10^5^ cells per well. Subsequently, the cells were incubated with PBS, NIR, CL, CEL, Cu_2-X_S, CL+NIR, and CEL+NIR ([Cu]: 15μg/mL, NIR: 1064 nm, 1 W/cm^2^, for 8 min) for 8 hours. Mitochondrial membrane potential (MMP) was detected using the JC-1 dye solution (Thermo Fisher Scientific) through CLSM. At high membrane potential, JC-1 molecules accumulate in the mitochondria and display red fluorescence. At low membrane potential, JC-1 exists as a monomer and emits green fluorescence. Changes in MMP were measured by the ratio of red to green fluorescence intensity.

Mitochondrial permeability transition pore (mPTP) dysfunction is also associated with the reduction of mitochondrial membrane potential (MMP). The mPTP level of various treatments were measured by mPTP assay kit. Following similar treatment protocols, the cells were gathered, washed and co-incubated with a mix of Calcein AM and CoCl_2_. The degree to which the mPTP had opened was measured via FCM.

**In vitro evaluation of the cuproptosis pathway**

CT26 cells were seeded in 6-well plates and cultured for 24 hours until they reached a density of 3×10^5^ cells/well. The CT26 cells were then treated with PBS, NIR, CL, CEL, Cu_2-X_S, CL+NIR, and CEL+NIR ([Cu]: 15μg/mL, NIR: 1064 nm, 1 W/cm^2^, for 8 min) for 8 hours. After treatment, the cells were washed three times with PBS for western blotting to detect the protein expression of FDX1 and LIAS. Furthermore, to evaluate DLAT aggregation in vitro, CT26 cells were seeded into confocal dishes for 24 h. The cells were then treated with PBS, NIR, CL, CEL, Cu_2-X_S, CL+NIR, and CEL+NIR at specific concentrations for 8 h. Subsequently, the cells were fixed with 4% paraformaldehyde, incubated with the DLAT antibody at 4℃ overnight, and then incubated with a secondary antibody at room temperature for 1 hour. The cells were stained with Mitotracker for 30 min, followed by DAPI for 10 min, and imaged using CLSM (FDX1, DLAT, ATP7B from Thermo Fisher Scientific; LIAS from Abcam plc).

**In vitro detection of immunogenic cell death (ICD) by CEL NP**

CT26 cells were prepared and treated with PBS, NIR, CL, CEL, Cu_2-X_S, CL+NIR, and CEL+NIR ([Cu]: 15μg/mL, NIR: 1064 nm, 1 W/cm^2^, for 8 min). 8 hours after incubation, the cells were fixed and blocked with Bovine Serum Albumin (BSA). The CT26 cells were then detected using a CRT antibody or HMGB1 antibody (Thermo Fisher Scientific) via CLSM. In addition, the supernatants from the treated cells were collected to evaluate ATP secretion using an ATP assay kit (Beijing Solarbio Science & Technology Co., Ltd., China). An ELISA assay was conducted to measure extracellular secretion of HMGB1 and cytokines TNF-α and IFN-γ in the extracellular milieu following various treatments.

**In vitro detection of Bone Marrow Dendritic Cells (BMDCs)**

First, BMDCs were isolated from the femurs and tibias of 3-4-week-old mice. Subsequently, CT26 cells were subjected to various treatment: PBS, NIR, CL, CEL, Cu_2-X_S, CL+NIR, and CEL+NIR ([Cu]: 15μg/mL, NIR: 1064 nm, 1 W/cm^2^, for 8 min). These treated CT26 cells were then co-incubated with BMDCs in a Transwell system for 24 hours. After the co-incubation period, BMDCs were collected, washed three times with PBS, and subsequently stained with anti-CD86 and anti-CD80. The samples were analyzed using a flow cytometry analysis to assess the activation status of BMDCs.

**Construction and applications of the CT26 tumor-bearing mouse model**

Three-week-old male BALB/c mice were purchased from the Laboratory Animal Center of Shantou University Medical College (SUMC). All experimental protocols were approved by the Animal Experimentation Ethics Committee of SUMC (SUMC2023-458). The formation of subcutaneous tumor models was based on previous published literature [2]. Prior to the experiment, the mice were allowed to acclimate to the housing conditions in a specific pathogen-free (SPF) environment for one to two weeks. The night before the subcutaneous cell injection, PBS and necessary tools (needles, syringes, pipette tips, and test tubes) were stored at a 4 ℃ refrigerator. CT26 cells (2×10^6^/100 μL) were digested, collected in PBS, and then subcutaneously injected into the left flank of the BALB/c mice to construct the tumor-bearing model. After injection, the mice were transferred to fresh cages and observed for 30 minutes ensure they returned to normal condition. Tumor length and width were recorded every three days using a vernier caliper, and tumor volume (TV) was calculated using the formula:

TV (mm^3^) = tumor length × tumor width^2^× 0.5.

**In vivo biodistribution of CEL NP**

To evaluate the tumor-targeting effect of the nanomaterials on tumor tissue, water-soluble indocyanine green (ICG) was loaded onto CEL NP as a near-infrared fluorescent dye. A dose of 100 μL CEL-ICG was injected into the CT26 tumor-bearing mice via the tail vein. The mice were anesthetized with Isoflurane, and fluorescence images were recorded using an In Vivo Imaging System (IVIS) (PerkinElmer, MA) at different time points (0, 1, 2, 4, 8, 12, 24, 48 hours). Twenty-four hours after treatment, the mice were sacrificed, and the major organs (liver, heart, spleen, lungs, kidneys) as well as tumor tissue were collected for ex vivo imaging.

**In vivo enhanced cuproptosis synergistical therapeutic effect of CEL NP**

The mice were randomly assigned to undergo the following treatments: PBS, NIR, CEL, Cu_2-X_S, CL+NIR, and CEL+NIR on days 1, 3 and 5. The “NIR” refers to irradiation with a NIR laser for 5 minutes (1064 nm, 1 W/cm^2^). During the treatments, temperature changes were monitored every minute using an infrared thermal camera (Fortric 600c). In addition, tumor volumes and body weights of the mice were recorded every 2 days. After two weeks, all mice were sacrificed for further analysis. The tumors were harvested and photographed, and tumor samples were collected for further analysis, including Ki-67, TUNEL, and H&E staining.

**In vivo biosafety evaluation of CEL NP**

The biosafety of CEL NP was assessed in CT26 tumor-bearing mice two weeks after treatment. Blood samples from each group were collected for hematological analysis, including the evaluation of aminotransferase (ALT), aspartate aminotransferase (AST), creatinine (CR), and blood urea nitrogen (BUN). Major organs (liver, heart, spleen, lungs, and kidneys) were also collected and subjected to H&E staining.

**In vivo assessment of infiltrating immune effects by CEL NP**

To analyze the antitumor immunity induced by CEL NP, primary tumor tissue was collected and digested in HBSS containing collagenase IV (0.3 mg/mL) at 37 ℃ for 1 hour. The digested tissue was then filtered through a 70 μm mesh to obtain a single-cell suspension. Subsequently, the cells were cultured with a live/dead Fixable Viability Dye. For dendritic cells (DCs), matured DCs in the lymph nodes were stained with anti-CD45, anti-CD11c, anti-CD80, and anti-CD86. For T cell infiltration analysis, cells from the primary tumors were stained with anti-CD45, anti-CD3, and anti-CD8 to evaluate the proportion of CD8^+^ T cells. Additionally, cells were stained with anti-CD45, anti-CD3, anti-CD4, and anti-Foxp3 to detect the content of regulatory T cells (Tregs). Finally, the proportion of all stained cells were analyzed using FCM.

**Statistical analysis**

The statistical analysis was performed using GraphPad Prism 8. Data were presented as mean ± standard deviation. One-way or Two-way analysis of variance (ANOVA) with Tukey’s post hoc test was used for multiple comparisons among more than two groups (column or grouped analyses). Survival analysis was conducted using the Kaplan‒Meier method, with statistical significance assessed via the log-rank test. A difference was considered statistically significant when the *p-*value was less than 0.05. The symbols *, **, ***, **** and ns were used to denote *p* < 0.05, *p* < 0.01, *p* < 0.001, *p* < 0.0001, and not significant, respectively.

**Supporting Contents:**


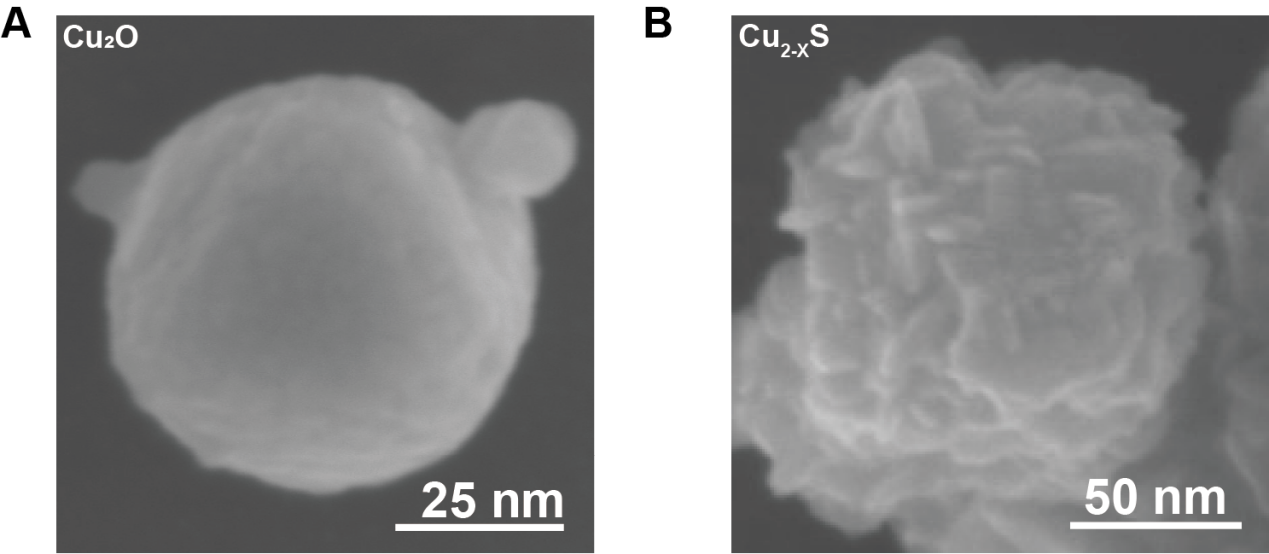


**Figure S1.** (A) SEM of Cu_2_O nanospheres (NSs); (B) Cu_2-X_S hollow nanospheres (HNSs)


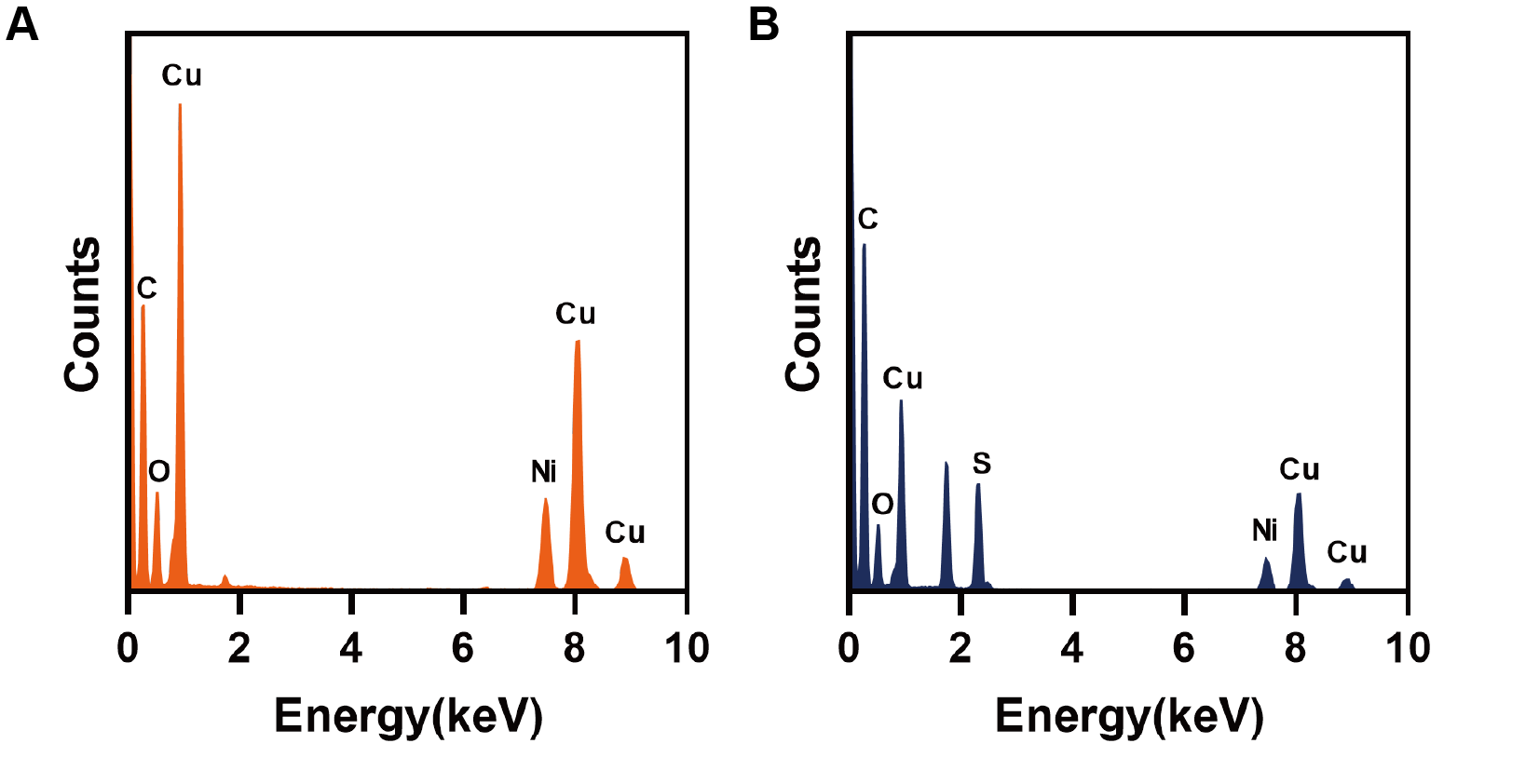


**Figure S2.** EDS of Cu_2_O NSs (A) and Cu_2-X_S HNSs (B).


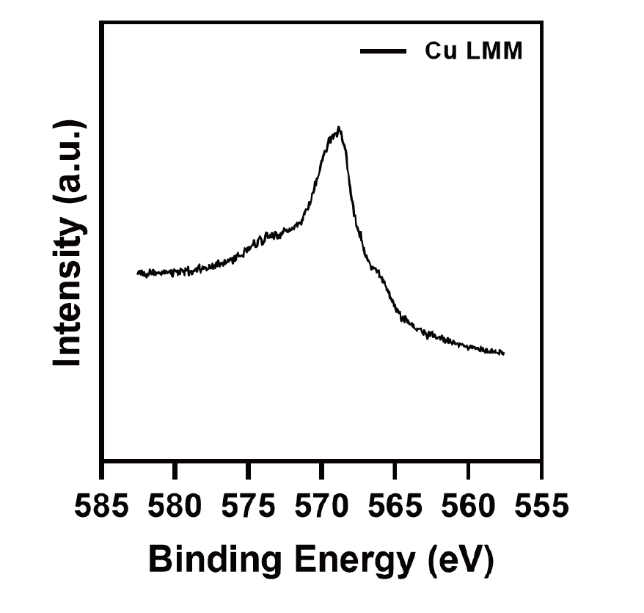


**Figure S3.** Cu LMM core-level XPS spectra of Cu_2-X_S HNSs


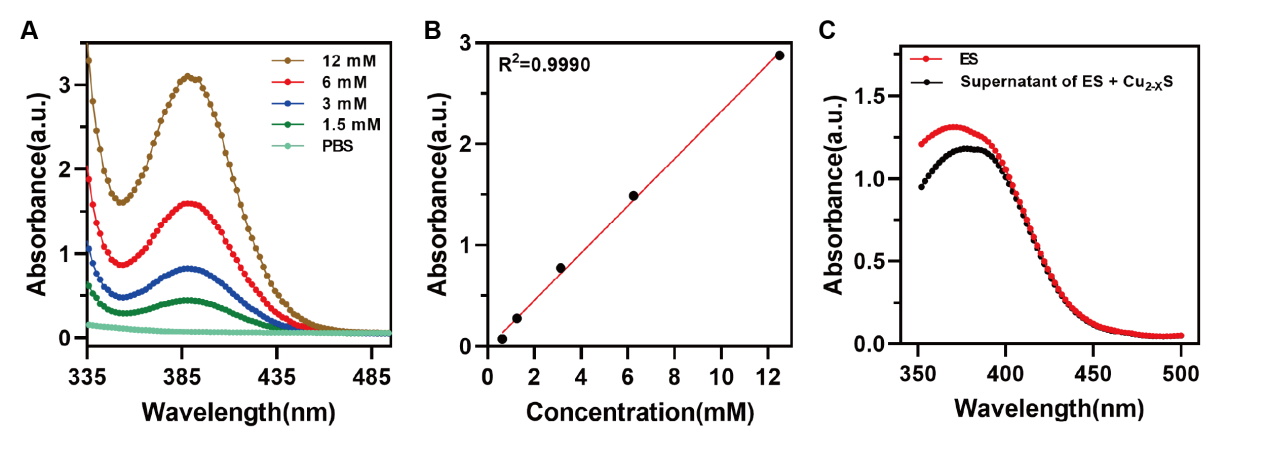


**Figure S4.** (A) UV-vis-NIR absorption spectra of ES at different concentration (0-12mM); (B) Absorbance of various ES concentrations at 380 nm. (C) UV-vis-NIR absorption spectra of 5 mM ES and the supernatant of the mixture containing Cu_2-X_S HNSs and ES.

**
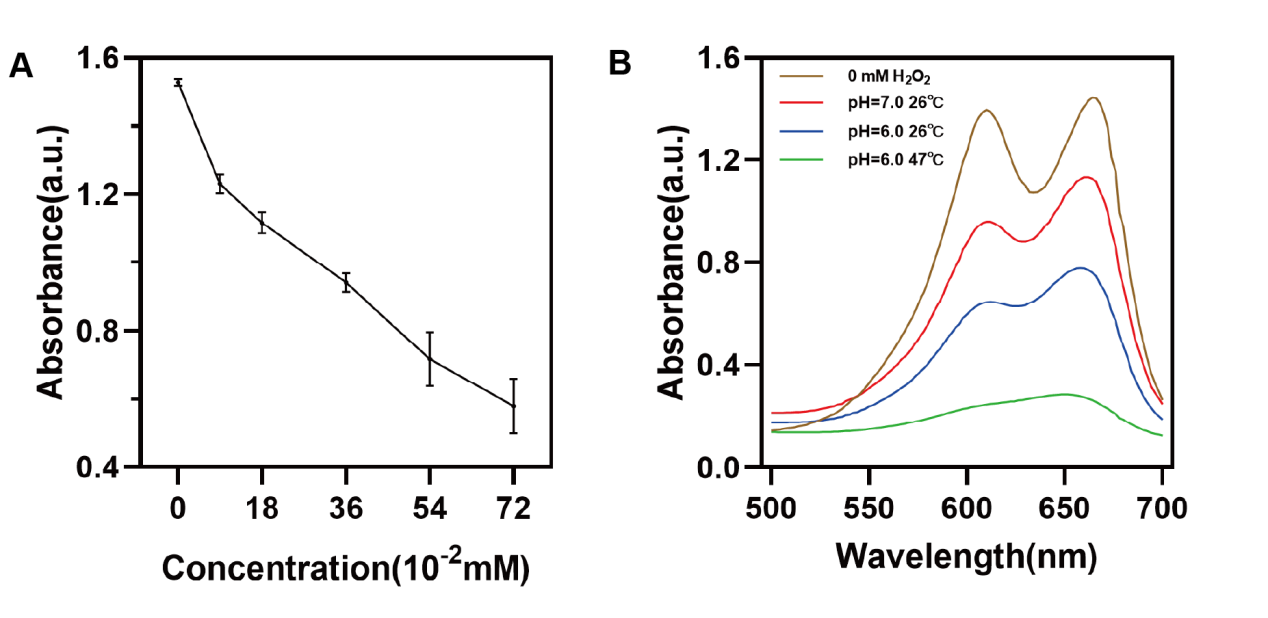
**

**Figure S5.** (A) statistics analysis of MB degeneration at different H_2_O_2_ concentrations caused by Cu_2-X_S HNSs (n = 5). (B) MB degradation under different experimental conditions. Data are presented as mean ± SD.


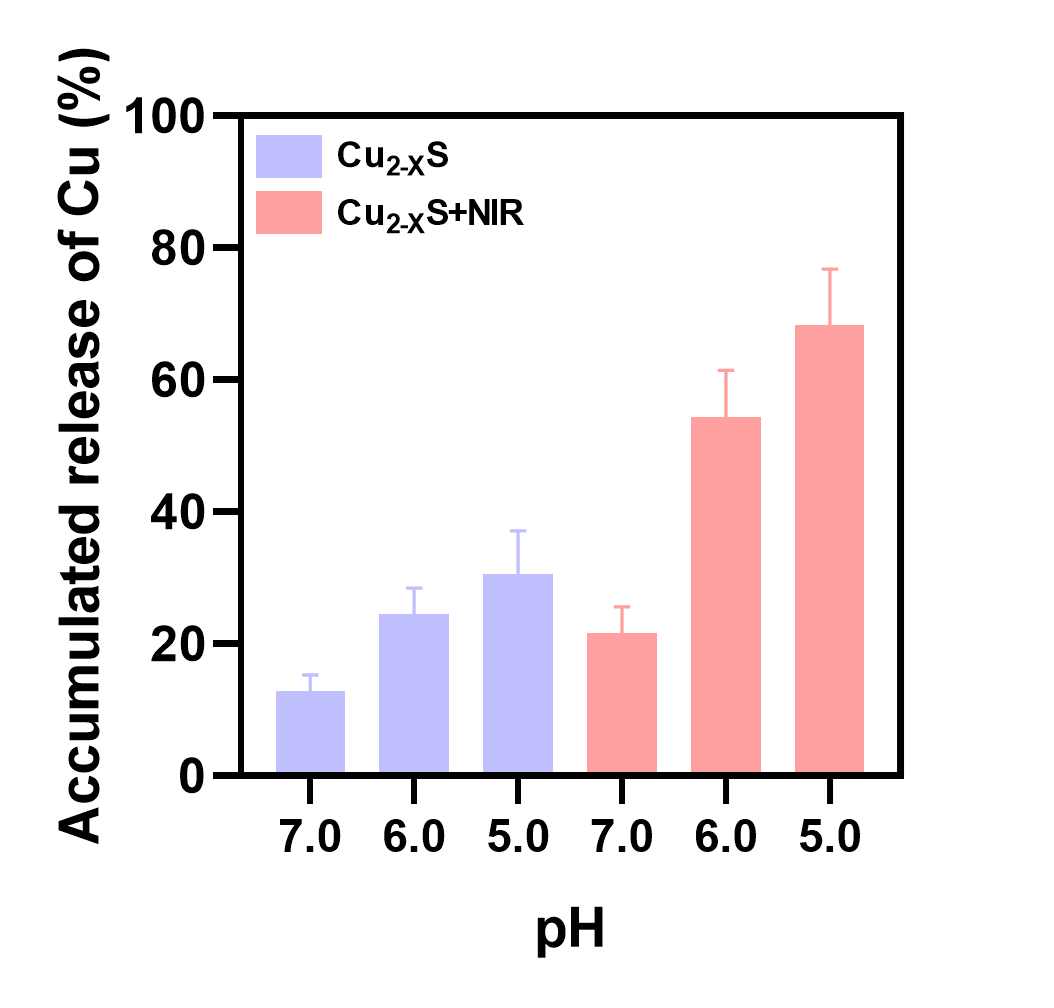


**Figure S6.** Release amount of copper ions from Cu_2-X_S HNSs solution with or without laser irradiation conditions in different pH values (under pH 5.0, 6.0 and 7.0) (n = 5). Data are presented as mean ± SD.


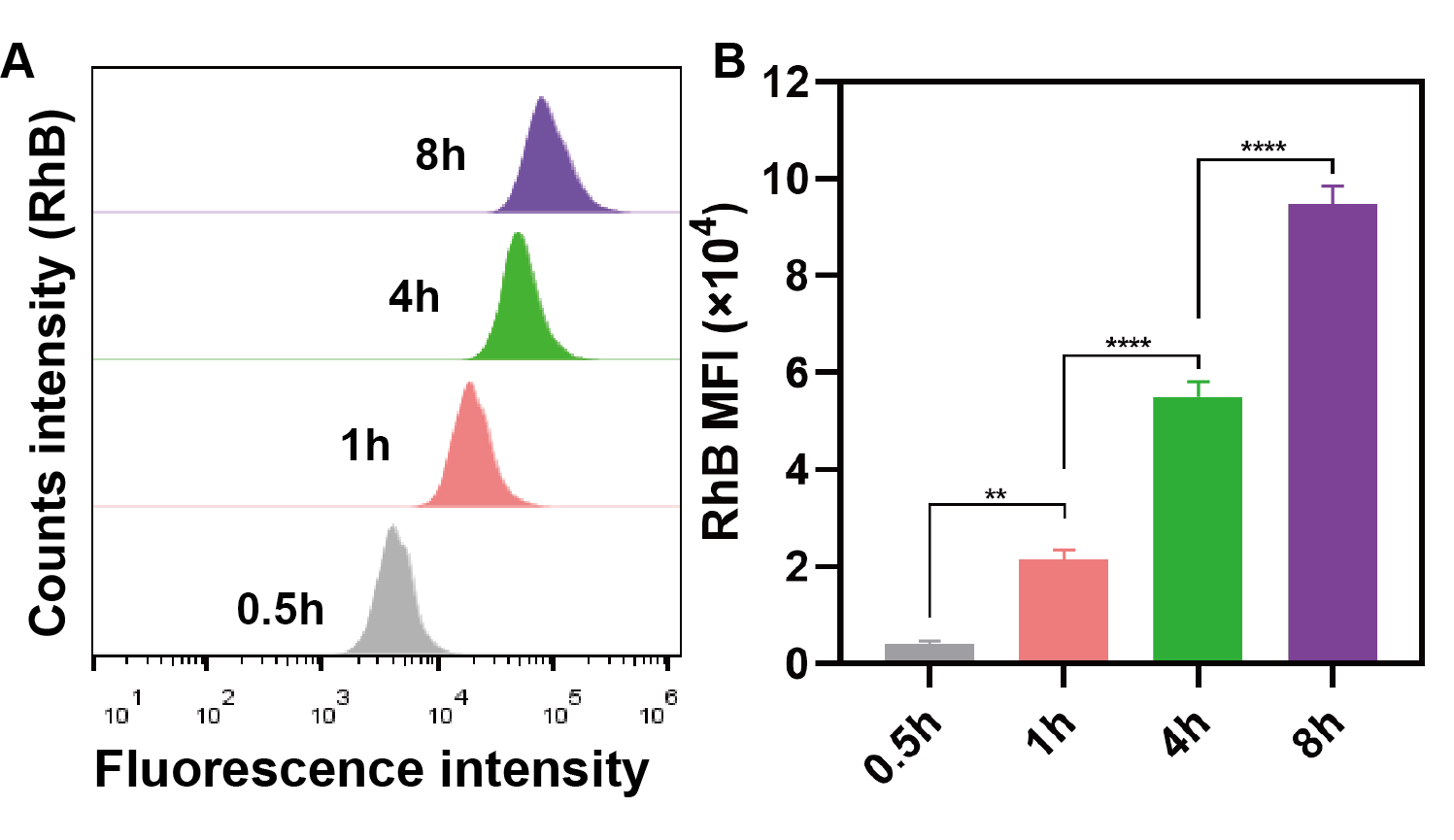


**Figure S7.** FCM analysis (A) and corresponding FCM quantification (B) of the intracellular uptake of CEL-RhB by CT26 cells. **p* < 0.05, ***p* < 0.01, ****p* < 0.001, *****p* < 0.0001, ns, not significant.

**
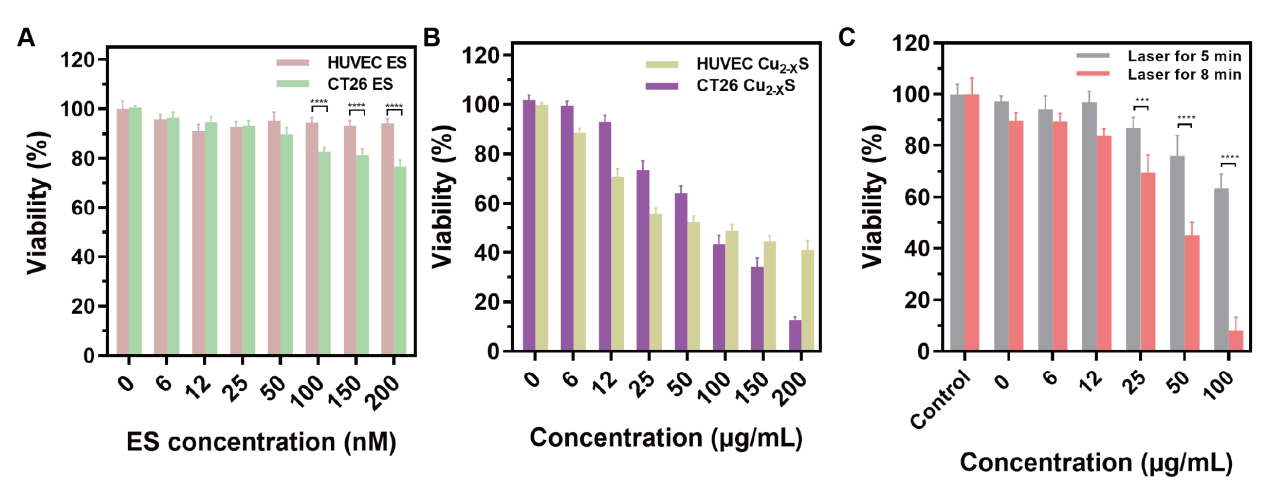
**

**Figure S8.** Relative viability of HUVEC and CT26 cells with different concentrations of ES (A) and bared Cu_2-X_S HNSs (B) (n = 5). (C) Viability of CT26 cells with different concentrations of CL and treated with irradiation (1064 nm, 1W/cm^2^) for 5 or 8 minutes (n = 5). Data are presented as mean ± SD. *p* values were calculated by one-way ANOVA test. **p* < 0.05, ***p* < 0.01, ****p* < 0.001, *****p* < 0.0001, ns, not significant.


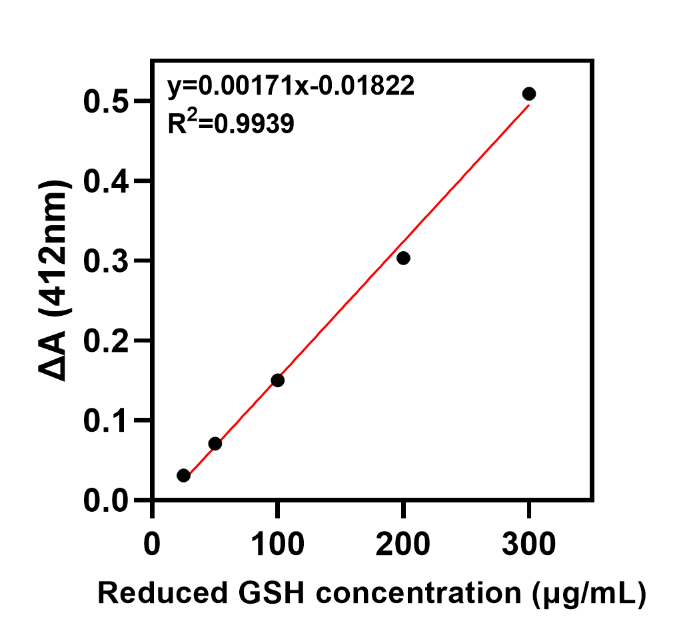


**Figure S9.** The linear relationship between several standard reduced glutathione (GSH) concentrations and Δ A at 412 nm.


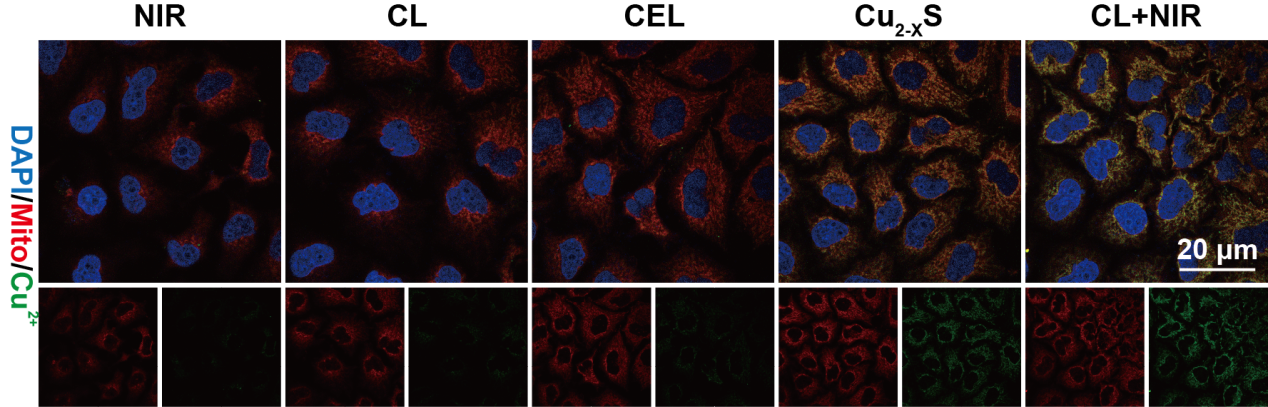


**Figure S10.** CLSM images of Cu^2+^ probe in CT26 cells after other groups of different treatments.


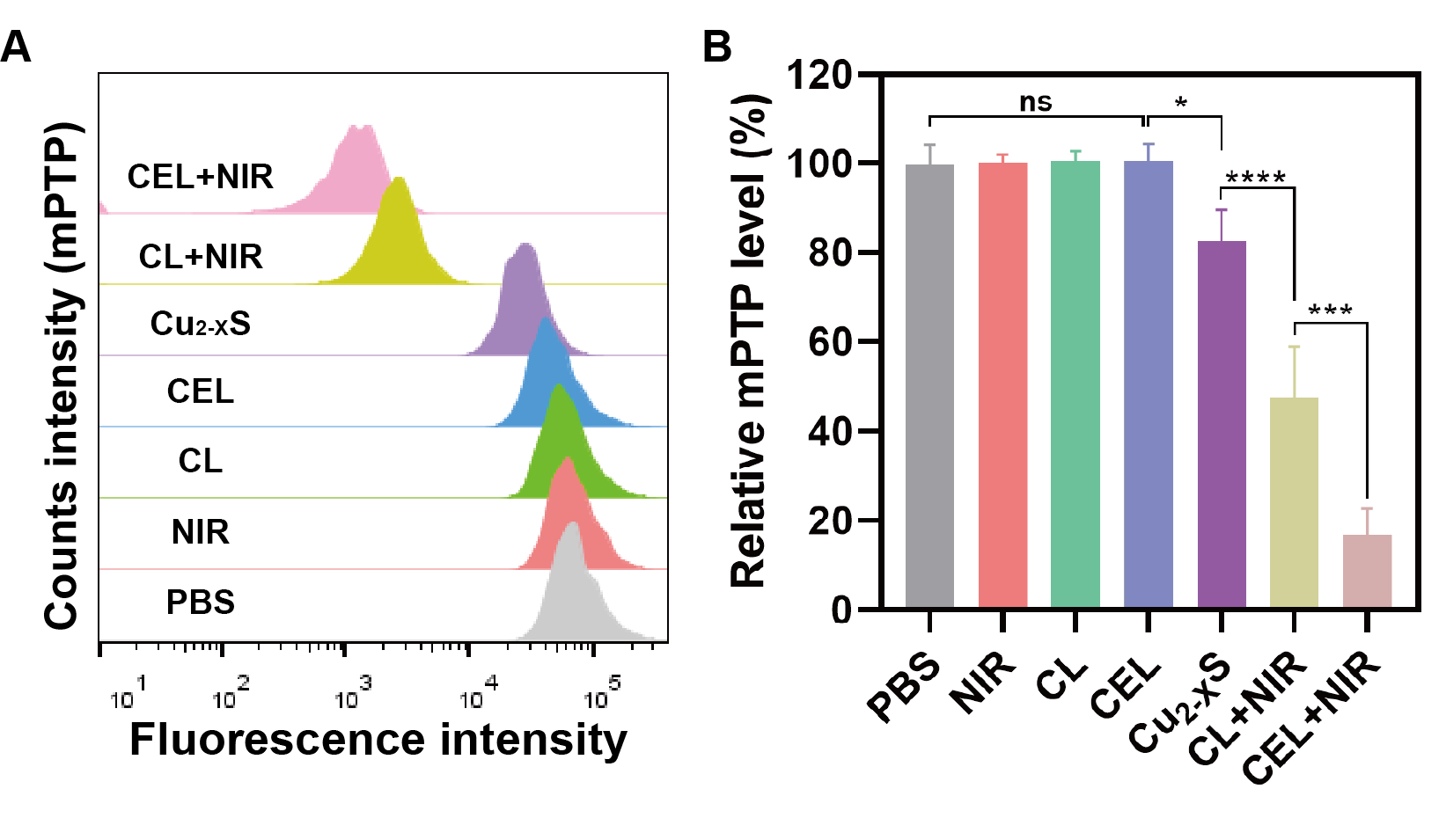


**Figure S11.** FCM analysis of the level of mPTP after several treatments. (n = 4). Data are presented as mean ± SD. *p* values were calculated by one-way ANOVA test. **p* < 0.05, ***p* < 0.01, ****p* < 0.001, *****p* < 0.0001, ns, not significant.

**
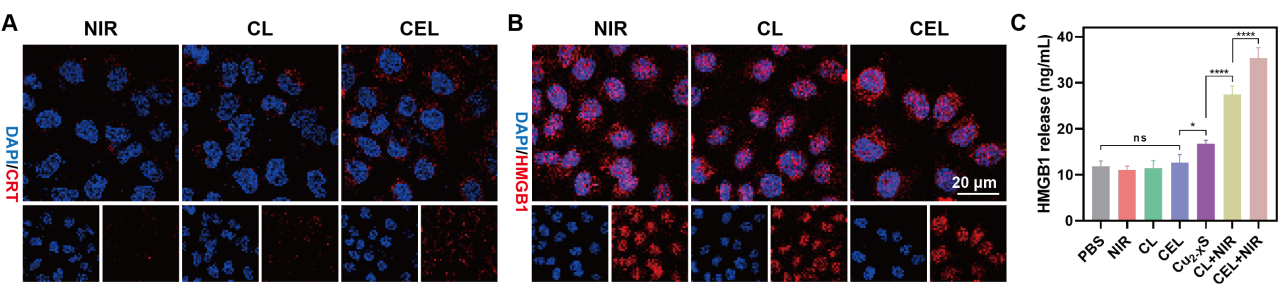
**

**Figure S12.** CLSM images of CRT (A) and HMGB1 (B) in CT26 cells after other treatments. (C) Quantification of the HMGB1 secretion within the medium of CT26 cells after various treatments (n = 5). Data are presented as mean ± SD. *p* values were calculated by one-way ANOVA test. **p* < 0.05, ***p* < 0.01, ****p* < 0.001, *****p* < 0.0001, ns, not significant.

**
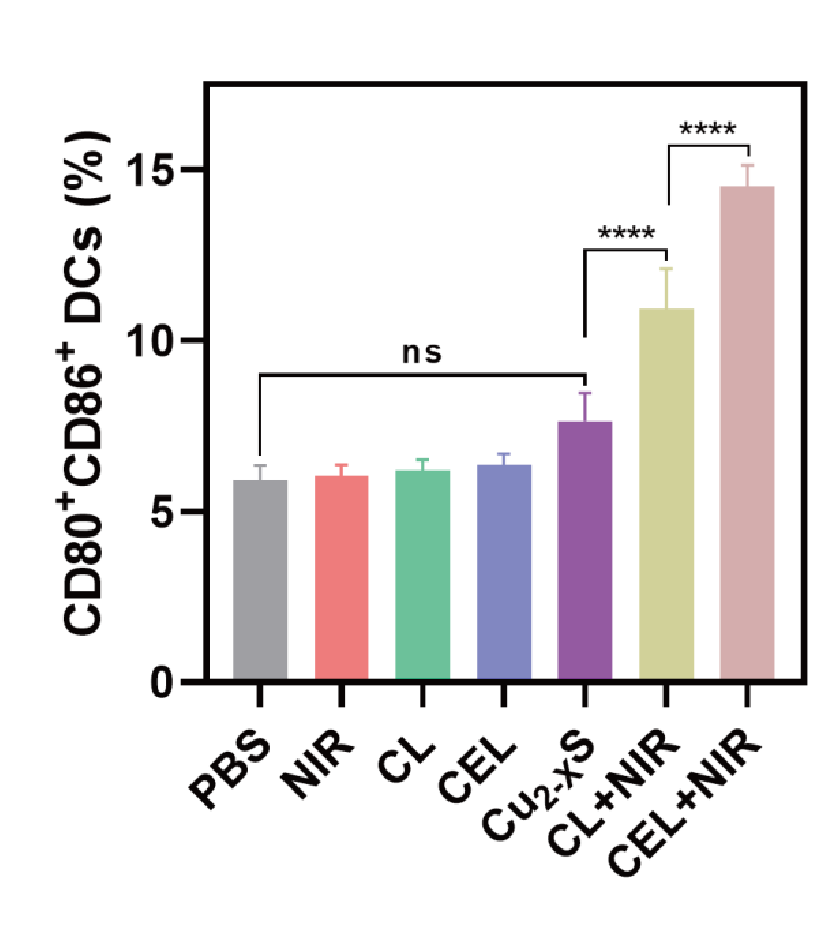
**

**Figure S13.** Corresponding quantification of the maturation of BMDCs upon various treatments (n = 4). Data are presented as mean ± SD. *p* values were calculated by one-way ANOVA test. **p* < 0.05, ***p* < 0.01, ****p* < 0.001, *****p* < 0.0001, ns, not significant.

**
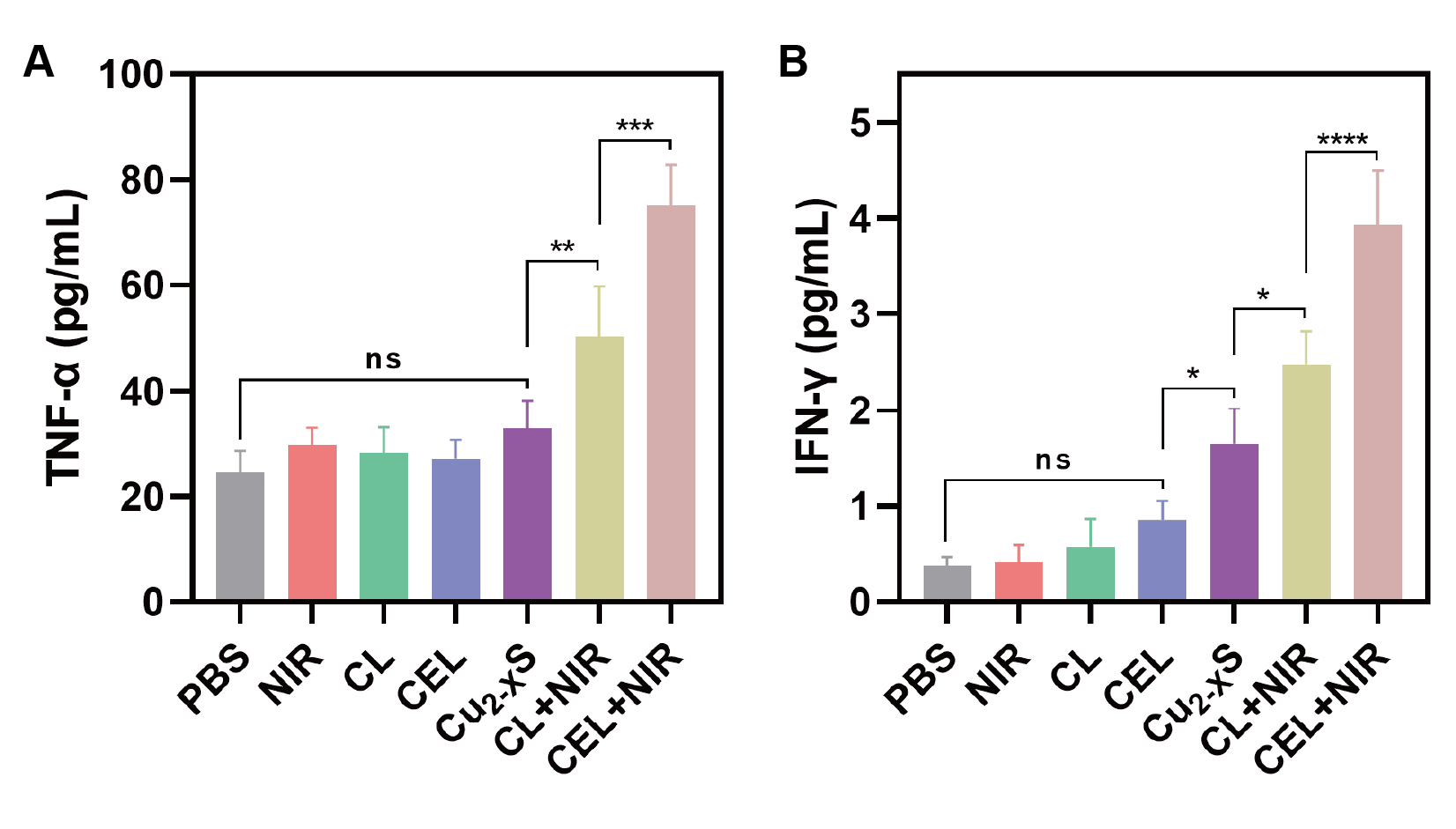
**

**Figure S14.** Cytokine contents of TNF-α (A) and IFN-γ (B) in the cellular medium of BMDCs following different treatments (n = 5). Data are presented as mean ± SD. *p* values were calculated by one-way ANOVA test. **p* < 0.05, ***p* < 0.01, ****p* < 0.001, *****p* < 0.0001, ns, not significant.


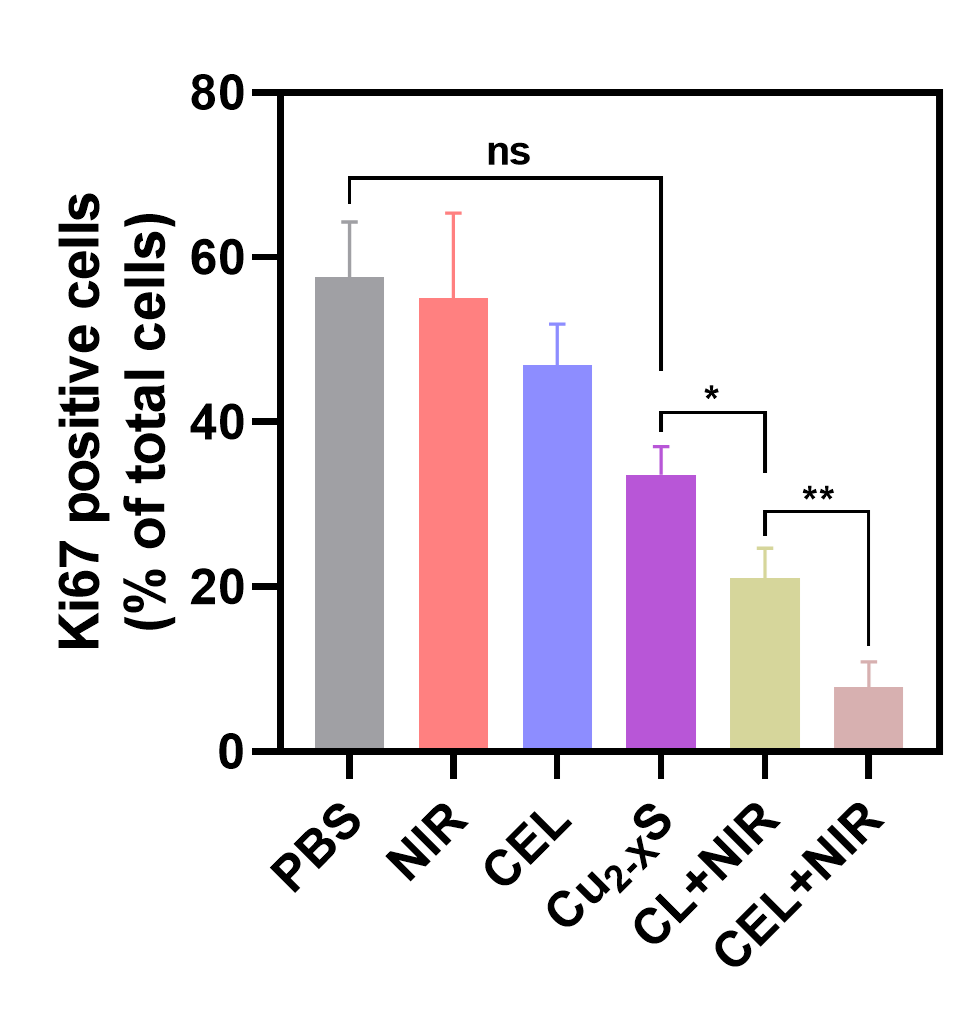


**Figure S15.** Counts of Ki67 positive cells in tumor tissues from mice underwent various treatments (n = 5). Data are presented as mean ± SD. *p* values were calculated by one-way ANOVA test. **p* < 0.05, ***p* < 0.01, ****p* < 0.001, *****p* < 0.0001, ns, not significant.


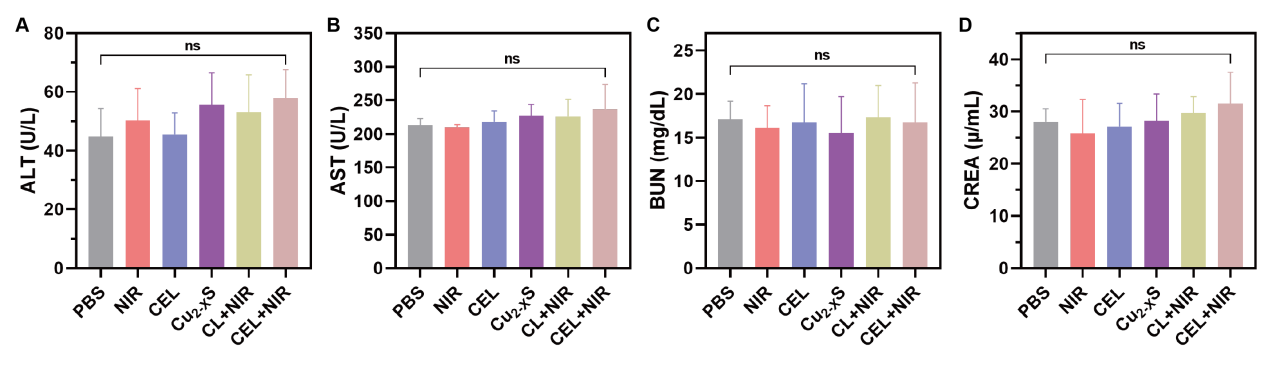


**Figure S16.** (A-B**)** Liver function indexes (alanine aminotransferase, ALT; aspartate aminotransferase, AST) of mice underwent various treatments. (C-D) Kidney function indexes (creatinine, CREA; blood urea nitrogen, BUN) of mice underwent various treatments (n = 5). Data are presented as mean ± SD. *p* values were calculated by one-way ANOVA test. **p* < 0.05, ***p* < 0.01, ****p* < 0.001, *****p* < 0.0001, ns, not significant.


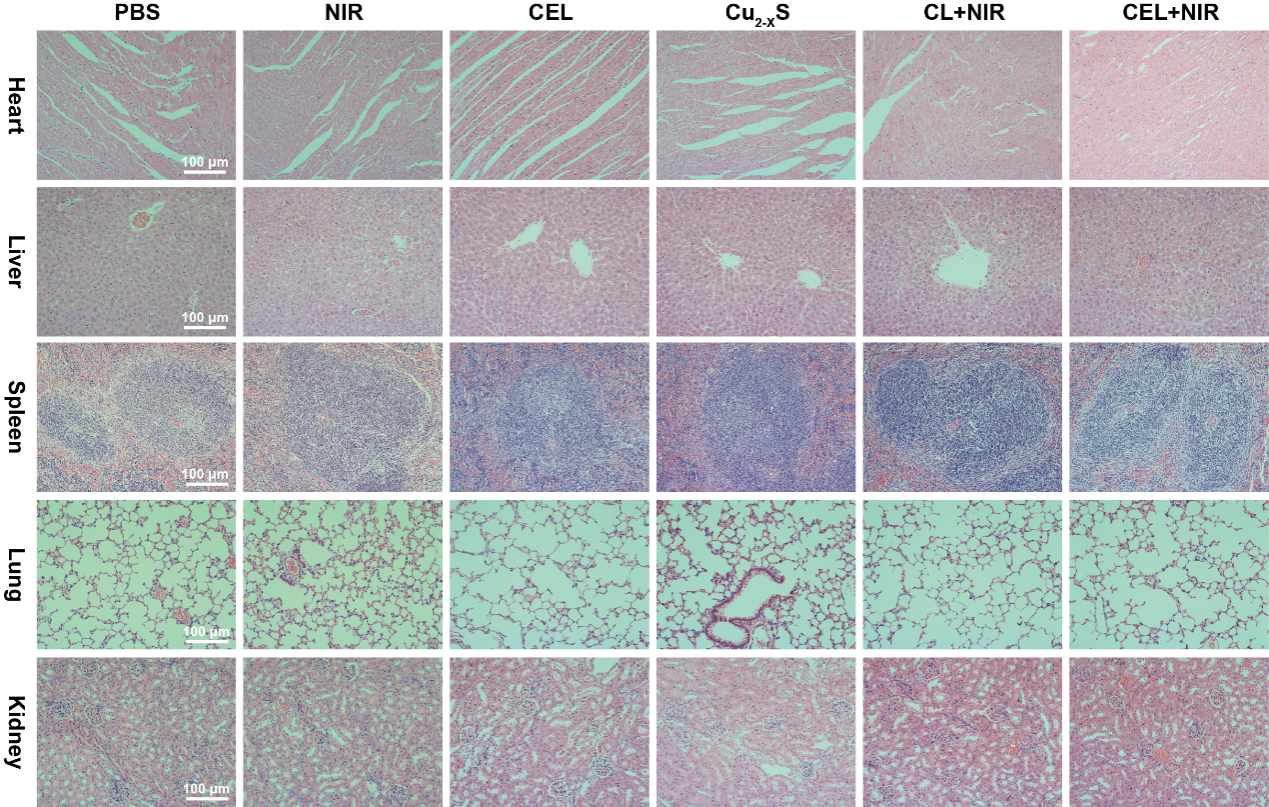


**Figure S17.** H&E staining of the major organs (heart, liver, spleen, lung, kidney) of the different groups.

Reference:

[1] D. K. Roper, W. Ahn, M. Hoepfner, *J Phys Chem C Nanomater Interfaces* **2007**, *111*, 3636.

[2] S. M. Stribbling, A. J. Ryan, *Nat Protoc* **2022**, *17*, 2108.
